# BTK kinase activity is dispensable for the survival of diffuse large B-cell lymphoma

**DOI:** 10.1016/j.jbc.2022.102555

**Published:** 2022-09-29

**Authors:** Hongwei Yuan, Yutong Zhu, Yalong Cheng, Junjie Hou, Fengjiao Jin, Menglin Li, Wei Jia, Zhenzhen Cheng, Haimei Xing, Mike Liu, Ting Han

**Affiliations:** 1 College of Life Sciences, Beijing Normal University, Beijing, China; 2 National Institute of Biological Sciences, Beijing, China; 3 BeiGene (Beijing) Co, Ltd, Beijing, China; 4 Deepkinase Co, Ltd, Beijing, China; 5 Tsinghua Institute of Multidisciplinary Biomedical Research, Tsinghua University, Beijing, China

**Keywords:** kinase-inactive BTK, DLBCL, phospho-tyrosine proteomics, PROTAC, TLR9, BCR, B cell receptor, BTK, Bruton's tyrosine kinase, BTKi, Inhibitors that target BTK, DLBCL, diffused large B-cell lymphoma, DPBS, Dulbecco's phosphate-buffered saline, PROTAC, proteolysis-targeting chimera

## Abstract

Inhibitors targeting Bruton's tyrosine kinase (BTK) have revolutionized the treatment for various B-cell malignancies but are limited by acquired resistance after prolonged treatment as a result of mutations in BTK. Here, by a combination of structural modeling, *in vitro* assays, and deep phospho-tyrosine proteomics, we demonstrated that four clinically observed BTK mutations—C481F, C481Y, C481R, and L528W—inactivated BTK kinase activity both *in vitro* and in diffused large B-cell lymphoma (DLBCL) cells. Paradoxically, we found that DLBCL cells harboring kinase-inactive BTK exhibited intact B cell receptor (BCR) signaling, unperturbed transcription, and optimal cellular growth. Moreover, we determined that DLBCL cells with kinase-inactive BTK remained addicted to BCR signaling and were thus sensitive to targeted BTK degradation by the proteolysis-targeting chimera. By performing parallel genome-wide CRISPR-Cas9 screening in DLBCL cells with WT or kinase-inactive BTK, we discovered that DLBCL cells with kinase-inactive BTK displayed increased dependence on Toll-like receptor 9 (TLR9) for their growth and/or survival. Our study demonstrates that the kinase activity of BTK is not essential for oncogenic BCR signaling and suggests that BTK’s noncatalytic function is sufficient to sustain the survival of DLBCL.

During B cell development, antigen-mediated cross-linking of the B cell receptor (BCR) activates the nonreceptor tyrosine kinases Bruton's tyrosine kinase (BTK) (1). BTK then promotes the activation of phospholipase Cγ2 (PLCγ2), which hydrolyzes phosphatidylinositol 4,5-bisphosphate to elicit an increase of intracellular Ca^2+^ (2, 3). The resulting Ca^2+^ flux activates diverse transcriptional programs to promote B cell proliferation and differentiation (4, 5). Oncogenic mutations, microbial antigens, or autoantigens can co-opt BCR signaling to support the growth and/or survival of malignant B cells, resulting in B-cell leukemias and lymphomas (6, 7, 8, 9, 10, 11). Inhibitors that target BTK (BTKi) have emerged as breakthrough therapies for treating a variety of B-cell malignancies, such as chronic lymphocytic leukemia/small lymphocytic lymphoma, mantle cell lymphoma, and Waldenström’s macroglobulinemia (12, 13, 14). However, durable response to BTKi is hampered by acquired resistance after prolonged treatment (15, 16).

First- and second-generation BTKi, such as ibrutinib, acalabrutinib, and zanubrutinib, inhibit the kinase activity of BTK by binding to its ATP-binding pocket and then covalently modifying a cysteine residue at position 481 (C481) of BTK (17, 18). Correspondingly, the most common mechanism of BTKi resistance occurs through mutations changing this reactive cysteine into serine (C481S) and less frequently into phenylalanine, tyrosine, or arginine (C481F, C481Y, and C481R) (19, 20). In addition, several non-C481 mutations have been observed in patients resistant to irreversible BTKi (19, 21) and, more recently, in relapsed chronic lymphocytic leukemia patients treated with the noncovalent BTK inhibitor pirtobrutinib (22). Whereas previous studies have clarified their mechanism of resistance to BTKi, whether these BTK mutations affect the biochemical activity and/or function of BTK in malignant B cells are not well understood.

## Results

### C481F/Y/R and L528W impair BTK kinase activity *in vitro*

**Figure 1:**
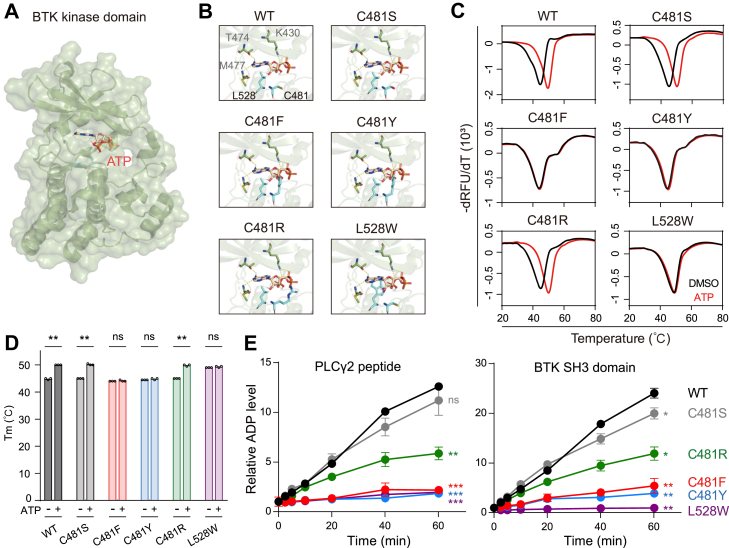
**C481F, C481Y, C481R, and L528W impair BTK kinase activity *in vitro*.***A*, a modeled structure of WT BTK kinase domain (PDB 6J6M) in complex with ATP. *B*, details of the modeled interface between the indicated BTK kinase domain and ATP. Key residues of BTK and ATP are shown as *sticks*. Carbon atoms of ATP are shown in *champagne*. Hydrogen bonds between BTK and ATP are shown as *yellow dotted lines*. *C*, thermal shift of recombinant BTK kinase domain in the presence of DMSO or 1 mM ATP from three technical replicates. *D*, melting temperature (Tm) quantification of data in (*C*). Data are the mean of three technical replicates. Significance was analyzed using Student’s *t* test, two-tail, paired (ns: not significant; ∗∗*p* < 0.01). *E*, *in vitro* kinase assay of recombinant BTK kinase domain using a synthesized PLCγ2 peptide (*left*) and recombinant BTK SH3 domain (*right*) as substrates. Data are the mean ± SD of three technical replicates. Comparisons between each mutant with WT BTK were analyzed using one-way ANOVA with Dunnett's multiple comparison tests (ns: not significant; ∗*p* < 0.05, ∗∗*p* < 0.01, ∗∗∗*p* < 0.001). BTK, Bruton's tyrosine kinase; DMSO, dimethyl sulfoxide.

In an attempt to explore the biochemical impact of clinically observed BTK mutations, we first docked ATP into the active site of BTK kinase domain. In the resulting structural model, the adenine ring of ATP formed hydrogen bonds with the side chain of T474 and the backbone of M477 in BTK. In addition, the phosphate group of ATP was locked in the active site of BTK by forming two hydrogen bonds with the side chain of K430. Residues C481 and L528 were located below the binding pocket and showed no direct interaction with ATP (Fig. 1, *A* and *B*). Next, we evaluated whether clinically observed BTK C481 and non-C481 mutations might affect ATP binding. Whereas C481S was not expected to affect the mode of ATP binding, substitutions of C481 by bulky side chains of phenylalanine (C481F), tyrosine (C481Y), or arginine (C481R) were all predicted to generate steric clashes to the sugar ring or the phosphate group of ATP (Fig. 1*B*). Similarly, leucine at position 528 mutated to tryptophan (L528W) caused a steric clash to the adenine ring of ATP (Fig. 1*B*). To verify these predictions, we purified recombinant BTK kinase domains and performed the thermal shift assay. We observed that ATP stabilized the kinase domains of BTK WT, C481S, and C481R but did not stabilize the kinase domains of BTK C481F, C481Y, and L528W (Fig. 1, *C* and *D*). Compared to phenylalanine and tyrosine, arginine may be more flexible at the active site due to the lack of an aromatic ring in its side chain. Thus, ATP may gain access to the active site and stabilize the kinase domain of BTK C481R.

These observations prompted us to examine the kinase activity of BTK mutants using an *in vitro* kinase assay. By incubating the BTK kinase domain with either a peptide substrate derived from PLCγ2 or a protein substrate (the SH3 domain of BTK containing an auto-phosphorylation site), we observed a near complete lack of kinase activity of BTK C481F, C481Y, and L528W *in vitro* (Fig. 1*E*). Notably, although C481R did not show a defect in ATP binding in the thermal shift assay, its kinase activity was significantly reduced compared with that of the WT BTK (Fig. 1*E*). Taken together, these results revealed that four clinically observed BTK mutations, C481F, C481Y, C481R, and L528W, impaired BTK kinase activity *in vitro*.

### C481F/Y/R and L528W impair BTK kinase activity in DLBCL cells

To examine whether C481F/Y/R and L528W impaired BTK kinase activity in malignant B cells, we selected TMD8 and OCI-LY10, two diffused large B-cell lymphoma (DLBCL) cell lines of the activated B-cell subtype (9), as our experimental models because of their sensitivity to ibrutinib (Fig. S1*A*). We used a lentiviral vector to stably express BTK WT, C481S/F/Y/R, or L528W in TMD8 and OCI-LY10 cells at levels near their endogenous BTK (Fig. S1*B*) and then examined their sensitivity to ibrutinib. Whereas expression of WT BTK did not alter the sensitivity of TMD8 or OCI-LY10 to ibrutinib, expression of BTK C481S/F/Y/R and L528W conferred resistance to ibrutinib in both cell lines (Fig. S1*C*). These isogenic cell lines, in which endogenous BTK was inactivated by ibrutinib, allowed us to examine the biochemical and functional sequelae of BTK mutations.

**Figure 2:**
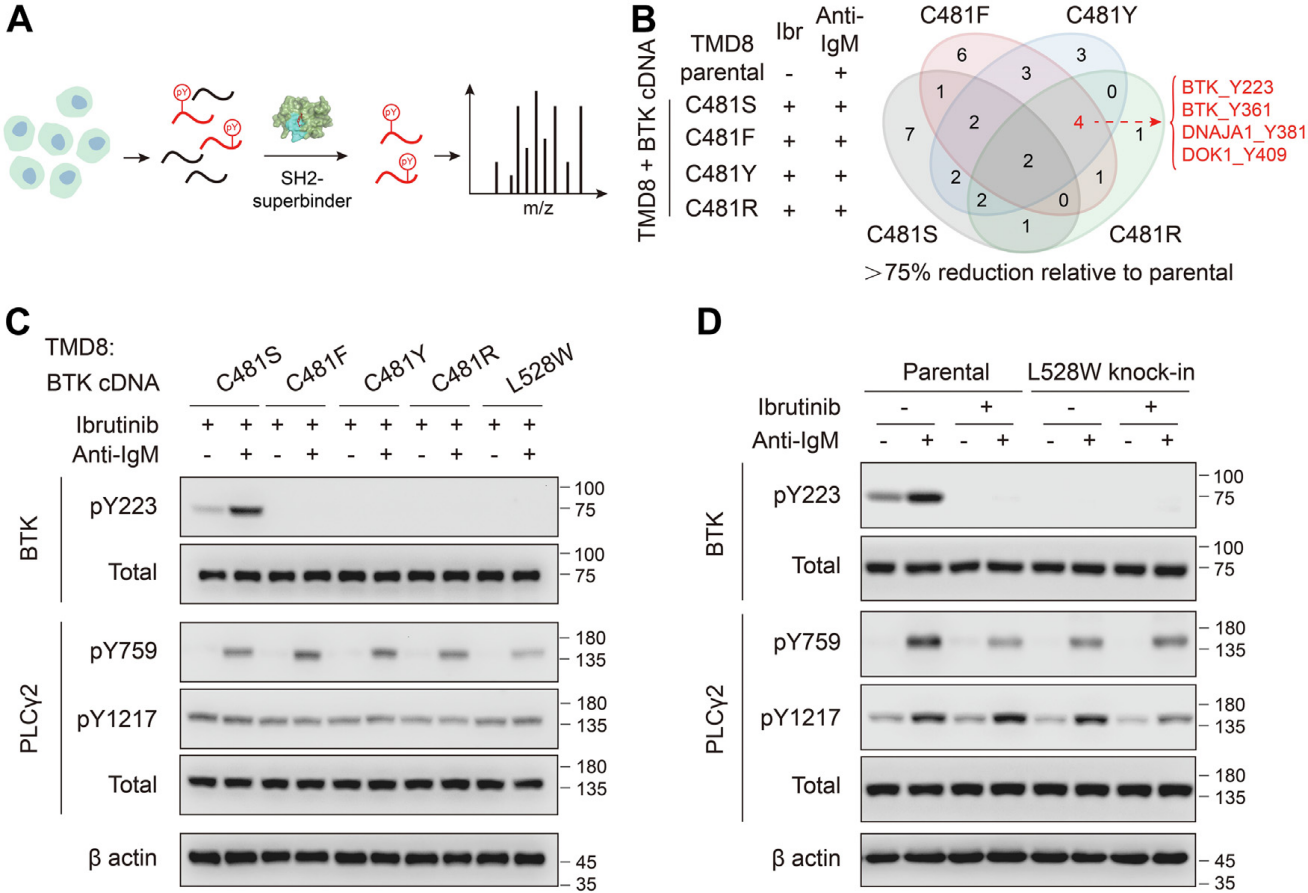
**BTK C481F, C481Y, C481R,and L528W are kinase-inactive in DLBCL cells.***A*, schematic of phospho-tyrosine (pY) proteomics. *B*, Venn diagram depicting the overlap of pY-modified peptides identified in parental TMD8 cells and TMD8 cells expressing BTK C481S/F/Y/R with indicated treatments. The four overlapping peptides in C481F/Y/R are listed. *C*, Western blotting of total and phosphorylated BTK and PLCγ2 in TMD8 cells expressing BTK C481S/F/Y/R and L528W with indicated treatments. *D*, Western blotting of total and phosphorylated BTK and PLCγ2 in parental and *BTK* L528W knock-in TMD8 cells with indicated treatments. Cells were pretreated with 10 nM ibrutinib for 6 h and then stimulated with 10 μg/ml goat antihuman IgM for 5 min. BTK, Bruton's tyrosine kinase; DLBCL, diffuse large B-cell lymphoma.

Because BTK is a tyrosine kinase, we used deep phospho-tyrosine (pY) proteomics to profile changes of global pY patterns in DLBCL cells following BTK inactivation by ibrutinib. After proteolytic digestion, pY-modified peptides were enriched by an Src homology 2-domain–derived pTyr superbinder (23) followed by identification by mass spectrometry (Fig. 2*A*). We identified 176 distinct pY-modified peptides from TMD8 and OCI-LY10 cells (Table S2). Two of these peptides, BTK pY223 and pY361, showed greater than 75% reduction in both cell lines following ibrutinib treatment (Fig. S2*A*). Moreover, we used anti-IgM stimulation to enhance BCR signaling, resulting in the identification of 218 distinct pY-modified peptides (Table S2). Five of these pY-modified peptides, BTK pY223, BTK pY361, CCDC50 pY146, ESYT1 pY822, and OCIAD1 pY199, showed greater than 75% reduction in both cell lines following ibrutinib treatment (Fig. S2*A*).

We next used deep pY proteomics to compare the global pY patterns of TMD8 cells expressing BTK C481S/F/Y/R (treated with ibrutinib to inactivate their endogenous BTK) with those of parental TMD8 cells. Among the pY-modified peptides that showed greater than 75% reduction relative to parental cells, four were common in C481F/Y/R, including BTK pY223, BTK pY361, DNAJA1 pY381, and DOK1 pY409 (Fig. 2*B*). BTK pY223 is a well-known auto-phosphorylation site of BTK (24). Under basal conditions, BTK Y223 phosphorylation could be detected in parental TMD8 and BTK C481S-expressing cells but was missing in TMD8 cells expressing BTK C481F/Y/R or L528W (Figs. 2*C* and S2*B*). Cross-linking of BCR by anti-IgM increased BTK Y223 phosphorylation in parental and BTK C481S-expressing TMD8 cells but not in BTK C481F/Y/R- or L528W-expressing TMD8 cells (Figs. 2*C* and S2*B*). Similar observations were made in OCI-LY10 cells (Fig. S2, *B* and *C*).

To further validate findings from deep pY proteomics, we employed CRISPR genome editing in TMD8 to obtain a *BTK* L528W knock-in clone, which was 264-fold less sensitive to ibrutinib than the parental TMD8 (Fig. S3, *A* and *B*). In contrast, the proliferation rate of *BTK* L528W knock-in clone was indistinguishable from that of parental TMD8 cells (Fig. S3*C*). Consistent with deep pY proteomics results, BTK Y223 phosphorylation was missing in *BTK* L528W knock-in TMD8 cells (Fig. 2*D*).

Activated BTK is known to phosphorylate PLCγ2 (Y753, Y759, and Y1217) (25). However, deep pY proteomics showed that PLCγ2 phosphorylation was not affected by the loss of BTK kinase activity (Table S2). Moreover, Western blotting confirmed the lack of correlation between PLCγ2 phosphorylation (pY759 and pY1217) and BTK kinase activity in both TMD8 and OCI-LY10 cells (Figs. 2*C* and S2, *B* and *C*). Similar observations were made in L528W knock-in TMD8 cells (Fig. 2*D*). Therefore, the identified pY sites of PLCγ2 are likely phosphorylated by a different kinase in DLBCL cells.

### Kinase-inactive BTK mutants support oncogenic BCR signaling in malignant B cells

**Figure 3:**
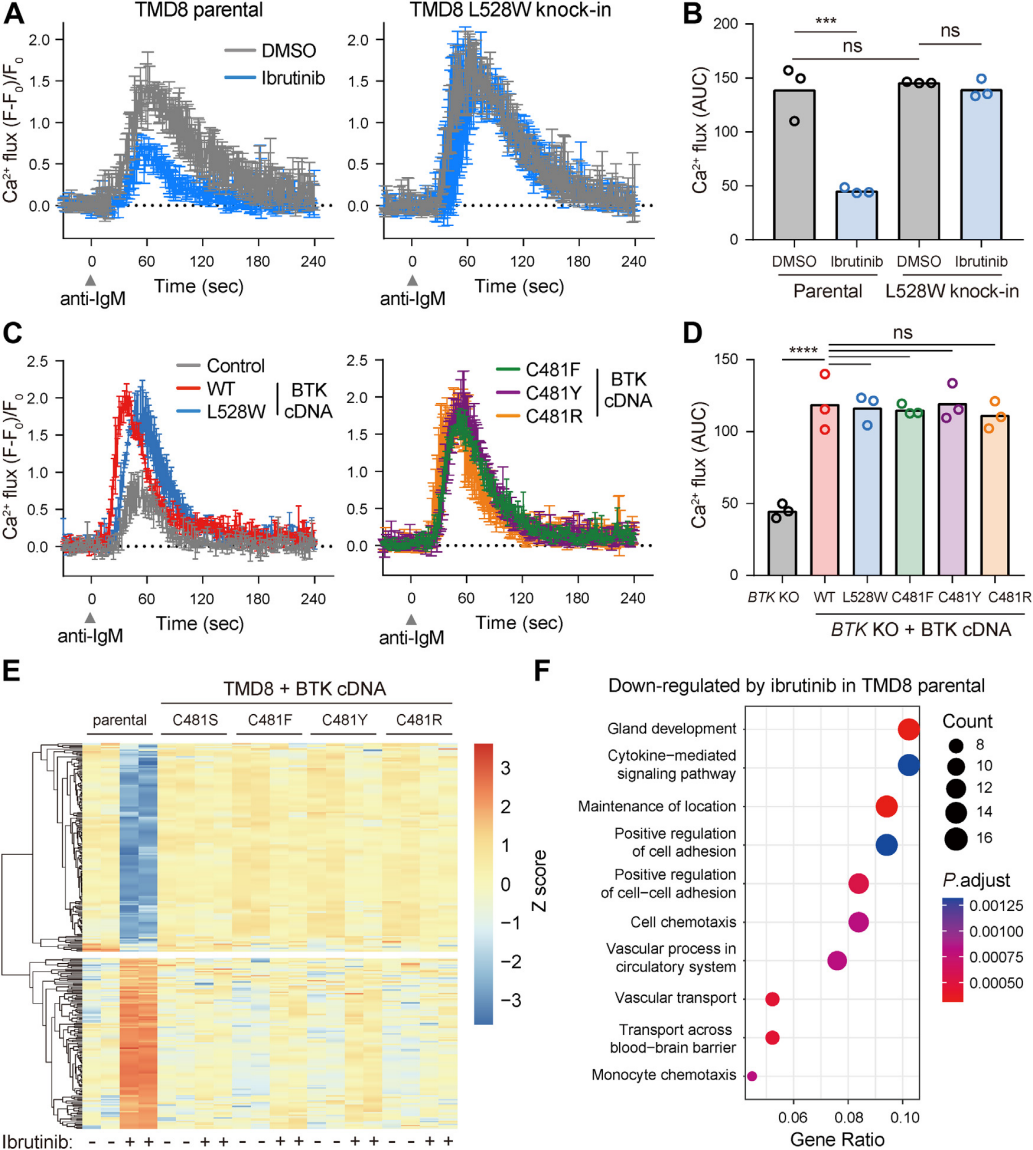
**Kinase-inactive BTK mutants support oncogenic BCR signaling in malignant B cells.***A*, anti-IgM–induced Ca^2+^ flux measurements in parental (*left*) and *BTK* L528W knock-in (*right*) TMD8 cells. Cells were pretreated with 10 nM ibrutinib for 12 h followed by 10 μg/ml anti-IgM stimulation. Data are the mean ± SD of three biological replicates. *B*, area under the curve (AUC) quantification of data in (*A*). Significance was analyzed using two-way ANOVA with Tukey’s multiple comparison tests (ns: not significant; ∗∗∗*p* < 0.001). *C*, anti-IgM–induced Ca^2+^ flux measurements in *BTK* KO Ramos cells expressing vector, BTK WT, C481F/Y/R, and L528W. Cells were stimulated with 10 μg/ml anti-IgM. Data are the mean ± SD of three biological replicates. *D*, area under the curve (AUC) quantification of data in (*C*). Significance was analyzed using one-way ANOVA with Tukey’s multiple comparison tests (ns: not significant; ∗∗∗∗*p* < 0.0001). *E*, heat map of differentially expressed genes in indicated cell lines with or without 10 nM ibrutinib treatment for 24 h. A row-wise Z score transformation was performed. *F*, gene ontology enrichment analysis of genes downregulated by ibrutinib in parental TMD8 cells. BCR, B cell receptor; BTK, Bruton's tyrosine kinase.

We next examined whether BTK kinase activity was required for oncogenic BCR signaling by measuring Ca^2+^ flux following BCR cross-linking. By testing a panel of B-cell lymphoma cell lines, we found that half of them displayed Ca^2+^ flux following anti-IgM stimulation (Fig. S4, *A* and *B*). Among the responding cell lines, TMD8 cells displayed Ca^2+^ flux peaked around 1 min and subsided by 4 min post anti-IgM stimulation. Pretreatment with ibrutinib reduced the magnitude of the Ca^2+^ flux in parental TMD8 cells by 3.2-fold (Fig. 3, *A* and *B*). Comparable anti-IgM–induced Ca^2+^ flux was observed in *BTK* L528W knock-in cells relative to parental cells, and the Ca^2+^ flux in *BTK* L528W knock-in cells could no longer be suppressed by ibrutinib (Fig. 3, *A* and *B*). These results suggest that TMD8 cells without BTK kinase activity maintain oncogenic BCR signaling.

Extending from findings in TMD8, we examined BCR signaling in the Burkitt’s lymphoma cell line Ramos, in which anti-IgM induced robust Ca^2+^ flux (Fig. S4, *A* and *B*). Ramos does not rely on BCR signaling for survival; we thus isolated a *BTK* KO clone of Ramos (Fig. S4*C*). The resulting *BTK* KO cells showed reduced Ca^2+^ flux following anti-IgM stimulation (Fig. S4*D*). We were initially surprised that knocking out *BTK* did not completely abrogate anti-IgM–induced Ca^2+^ flux; however, similar phenomena were observed in multiple previous studies (26, 27), suggesting that BTK-independent mechanisms could contribute to residual Ca^2+^ flux in *BTK* KO cells. We then expressed various BTK mutants and found all of them rescued the defective Ca^2+^ flux in *BTK* KO Ramos cells to the same degree as WT BTK (Fig. 3, *C* and *D*). These results altogether suggest that the kinase activity of BTK is dispensable for the induction of Ca^2^^+^ flux to transmit oncogenic BCR signaling.

### Kinase-inactive BTK does not alter gene expression in malignant B cells

BCR signaling activates multiple transcription factors to sustain the growth and survival of malignant B cells (4, 5). We therefore used RNA-seq to examine whether the loss of BTK kinase activity might affect gene expression downstream of BCR signaling (Table S3). By comparing parental TMD8 cells treated with vehicle or ibrutinib, we defined a list of 179 significantly upregulated genes and 192 significantly downregulated genes in ibrutinib-treated cells (Figs. 3*E* and S5*A*). Gene ontology analyses revealed that ibrutinib treatment downregulated genes involved in cytokine-mediated signaling pathways and regulation of cell adhesion and chemotaxis, consistent with previous studies (Fig. 3*F*) (28, 29). Genes upregulated by ibrutinib did not enrich gene sets with statistical significance (Fig. S5*F*). By displaying these differentially expressed genes as a heat map, we found that they were expressed at comparable levels in TMD8 cells expressing kinase-active BTK C481S or kinase-inactive BTK C481F/Y/R (Figs. 3*E* and S5, *B*–*E*). These results demonstrate that loss of BTK kinase activity does not affect gene expression downstream of BCR signaling.

### BTK kinase-inactivating mutations do not bypass BCR signaling

**Figure 4:**
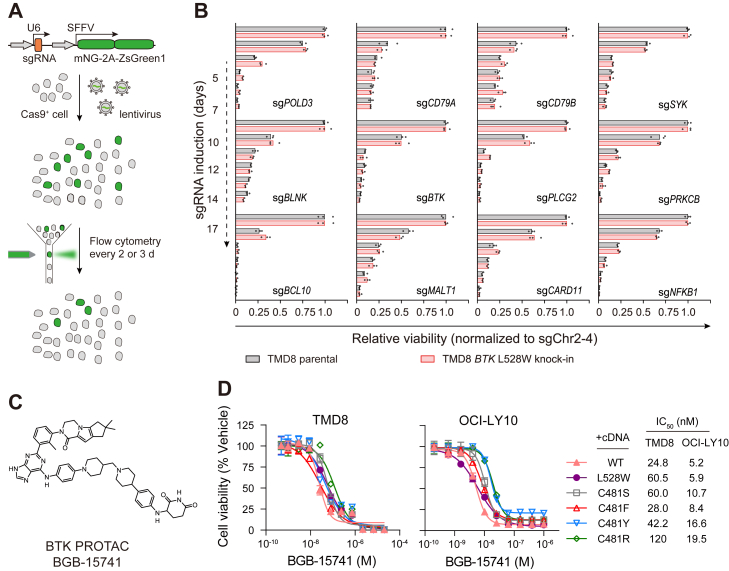
**DLBCL cells with kinase-inactive BTK maintain dependence on BCR signaling for survival.***A*, schematic of the competitive cell growth assay. Cas9-transduced cells were infected with lentivirus expressing an sgRNA and a fluorescent protein marker at a low multiplicity of infection (MOI = 0.2–0.4). Flow cytometry was used to monitor the percentages of transduced GFP^+^ cells every 2 or 3 days. *B*, viability effects (normalized to the control sgChr2-4) after CRISPR inactivation of BCR signaling–related genes in indicated cells. Data are the mean of three technical replicates. *C*, chemical structure of BGB-15741. *D*, BGB-15741 cytotoxicity measurements on TMD8 (*left*) and OCI-LY10 cells (*right*) expressing BTK WT, C481S/F/Y/R, and L528W. Data are the mean ± SD of three biological replicates. BCR, B cell receptor; BTK, Bruton's tyrosine kinase; DLBCL, diffuse large B-cell lymphoma.

We used a competitive cell growth assay to examine whether DLBCL cells deficient in BTK kinase activity remained dependent on BCR signaling for growth and survival (Fig. 4*A*). To ensure comparable CRISPR efficiencies, we isolated Cas9-transduced clones of parental TMD8 and *BTK* L528W knock-in cells and validated their dependencies on the pan-essential gene *POLD3* (Fig. S6*A*). We then used an sgRNA-targeting *BTK* and found that sgRNA-transduced cells from both parental and *BTK* L528W knock-in cells were depleted at comparable rates (Fig. 4*B*). The reduction of cell fitness due to loss of *BTK* could be fully rescued by complementary DNAs encoding either kinase-active BTK (WT and C481S) or kinase-inactive BTK (L528W and C481F/Y/R) (Fig. S6*B*).

We next transduced cells with sgRNAs that target several key genes of the BCR signaling pathway, including *CD79A*/*CD79B* (encoding components of the BCR complex), *SYK*, *BLNK* (encoding B cell linker protein), *PLCG2*, *PRKCB* (encoding protein kinase C β), *CARD11*/*BCL10*/*MALT1* (encoding components of the CBM signalosome complex), and *NFKB1* (encoding a nuclear factor kappa B subunit). We observed that both TMD8 parental cells and *BTK* L528W knock-in cells were equally dependent on these genes for survival (Fig. 4*B*). Altogether, these results demonstrate that DLBCL harboring BTK kinase-inactivating mutations does not bypass BCR signaling for growth and survival.

### Targeted degradation of BTK kinase-inactive mutants overcomes BTKi resistance

Because DLBCL cells with kinase-inactive BTK remained addicted to BTK, we hypothesized that targeted BTK degradation by the proteolysis-targeting chimera (PROTAC) might be an effective strategy to overcome BTKi resistance (30). Thus, we employed a BTK PROTAC (BGB-15741) to hijack the E3 ubiquitin ligase CRL4^CRBN^ to degrade BTK (Fig. 4*C*). BGB-15741 treatment induced the degradation of both WT BTK and BTK L528W in TMD8 cells, which could be blocked by the proteasome inhibitor MG132 and the CRBN binder lenalidomide (Fig. S7, *A* and *B*). TMD8 and OCI-LY10 cells harboring kinase-inactive BTK mutations were sensitive to the antiproliferative effect of BGB-15741 (Fig. 4*D*). Using quantitative mass spectrometry, we examined the proteome-wide selectivity of BGB-15741. In TMD8 cells treated with BGB-15741 for 6 h, BTK and JUN were the only two proteins significantly depleted (Fig. S7*C* and Table S4). By querying previous genome-wide CRISPR/Cas9 screening in TMD8 (31), we found that JUN was not essential for the survival of TMD8 (Fig. S7*D*). Thus, we conclude that the antiproliferative activity of BGB-15741 is a result of BTK degradation. Taken together, the BTK PROTAC BGB-15741 promotes the degradation of kinase-inactive BTK mutants to overcome resistance to irreversible BTKi.

### BTK kinase-inactivating mutations increase *TLR9* dependency in DLBCL cells

**Figure 5:**
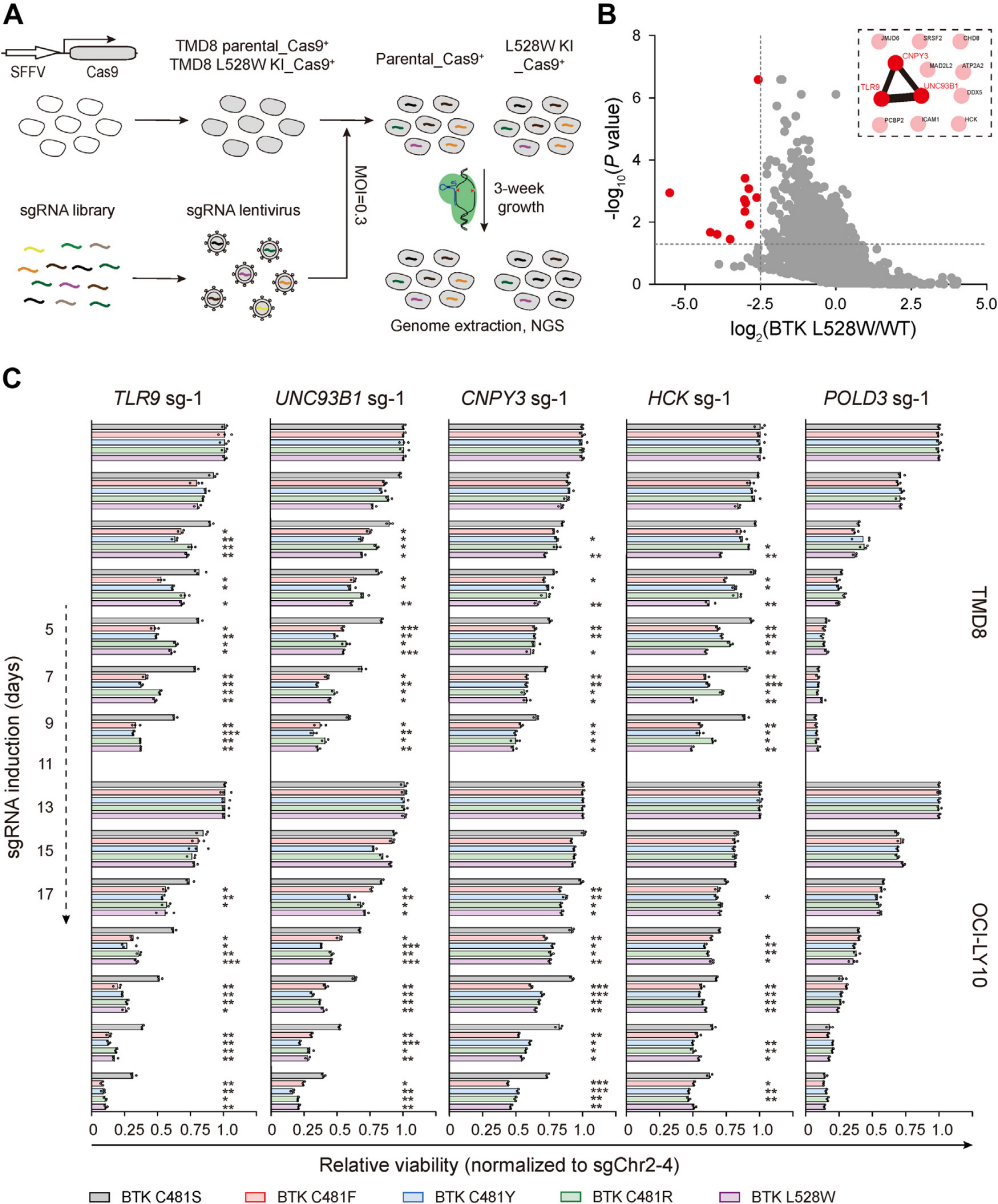
**Altered genetic dependencies of DLBCL cells with kinase-inactive BTK.***A*, schematic of parallel CRISPR-Cas9 screening in parental and *BTK* L528W knock-in TMD8 cells. *B*, scatterplot depicting log_2_-transformed average fold change of sgRNA abundance (*BTK* L528W knock-in divided by parental) and −log_10_ transformed *p*-value computed by MAGeCK. Functional association of genes identified in the CRISPR-Cas9 screen by STRING analysis is shown as an *inset*. *C*, viability effects (normalized to the control sgChr2-4) after CRISPR inactivation of the indicated genes in the indicated cell lines. Endogenous BTK of these cell lines were inactivated by ibrutinib. Data are the mean of three technical replicates from one representative experiment. Two independent experiments were performed. Significance was analyzed using one-way ANOVA with Dunnett's multiple comparison tests (∗*p* < 0.05, ∗∗*p* < 0.01, ∗∗∗*p* < 0.001). BTK, Bruton's tyrosine kinase; DLBCL, diffuse large B-cell lymphoma.

To unbiasedly explore whether BTK kinase-inactivating mutations might result in alterations of DLBCL genetic dependencies, we performed a parallel genome-wide CRISPR-Cas9 screening in parental TMD8 and L528W knock-in cells. After lentiviral transduction of the sgRNA library, we propagated cells for 3 weeks and then performed next-generation sequencing to quantify the abundance of each sgRNA in surviving cells (Fig. 5*A*).

MAGeCK (Model-based Analysis of Genome-wide CRISPR/Cas9 Knockout) algorithm ranked 12 significantly depleted genes in L528W knock-in cells relative to parental cells (Fig. 5*B* and Table S5). Among these 12 candidates, STRING analysis revealed that Toll-like receptor 9 (TLR9), unc-93 homolog B1 (UNC93B1), and canopy homolog 3 (CNPY3) formed a protein–protein interaction network (Fig. 5*B*). TLR9 is a member of the Toll-like receptor (TLR), which localizes to the endosomes and senses microbial DNA to trigger proinflammatory signaling (32). CNPY3 and UNC93B1 are required for proper TLR9 folding and localization to endosomes, respectively (33, 34, 35). In addition, sgRNAs targeting the nonreceptor tyrosine kinase HCK were also depleted in L528W knock-in cells relative to parental cells (Fig. 5*B*).

We next used the competitive cell growth assay (Fig. 4*A*) to validate findings from the CRISPR-Cas9 screening. When transduced with sgRNAs targeting *TLR9*, *BTK* L528W knock-in cells were depleted with significantly faster kinetics relative to parental cells (Fig. S8*A*). To exclude clonal effects in the competitive cell growth assay, we used the suite of isogenic TMD8-Cas9 cells expressing BTK C481S/F/Y/R and L528W and inactivated their endogenous BTK with ibrutinib. When transduced with the sgRNA-targeting *POLD3*, TMD8-Cas9 cells expressing different forms of BTK were depleted with similar kinetics, suggesting their comparable CRISPR efficiencies (Fig. 5*C*). In contrast, when transduced with sgRNAs targeting *TLR9*, *UNC93B1*, *CNYP3*, or *HCK*, BTK kinase-inactive cells (C481F/Y/R and L528W) were depleted with significantly faster kinetics than BTK kinase-active cells (C481S). (Figs. 5*C* and S8*B*). Similar observations were made in OCI-LY10-Cas9 cells (Figs. 5*C* and S8*B*). Taken together, we conclude that DLBCL cells with kinase-inactive BTK are more dependent on TLR9 signaling for their growth and/or survival.

## Discussion

Our study unveiled a collection of clinically observed BTK mutations that not only cause resistance to irreversible BTKi but also inactivate the kinase activity of BTK. These mutations affect two residues in BTK, C481, and L528, substituting them with residues containing bulky side chains, resulting in steric hindrance to ATP binding. By studying the biochemical and functional properties of these BTK mutants in DLBCL cell lines, we made the unexpected finding that kinase-inactive BTK mutants were as efficient at transducing BCR signaling as their WT counterpart. Similar observations of kinase-inactive BTK mutants have been made in other experimental systems. Tomlinson *et al.* (36) observed that a kinase-inactive BTK (K430E) could restore BCR-induced calcium flux and ERK-MAPK activation in *BTK*-deficient DT40 cells. In addition, BTK C481F and C481Y mutants have been reported to lack auto-phosphorylation activity in HEK 293T and DT40 cells (20, 27). Together with our results, BTK’s noncatalytic activity instead of kinase activity is required for oncogenic BCR signaling to support the growth and survival of malignant B cells.

Although protein kinases are known primarily as enzymes catalyzing phosphorylation, accumulating evidence has revealed their noncatalytic functions, such as allosteric regulation of other enzymes, scaffolding the assembly of signaling complexes, and regulation of transcription (37). Our parallel CRISPR-Cas9 screening in DLBCL cells with kinase-active *versus* kinase-inactive BTK revealed an increased genetic dependency of BTK kinase-inactive cells on *TLR9*, *UNC93B1*, *CNPY3*, and *HCK*. Thus, inactivation of BTK kinase activity resulted in a loss of cellular fitness only when these genes were inactivated.

HCK is a member of the SRC family of cytoplasmic tyrosine kinases (SFKs) and is expressed in myeloid cells and B lymphocytes (38). High levels of HCK have been reported in various types of leukemia, such as multiple myeloma and acute lymphoblastic leukemia (39, 40). In our study, we demonstrated that DLBCL cells with kinase-inactive BTK displayed increased genetic dependence on *HCK* compared to DLBCL cells with kinase-active BTK. Consistent with our finding, a recent study implicated the activation of HCK in malignant B cells with kinase-inactive BTK (27). Functions and mechanisms of HCK activation in DLBCL cells with kinase-inactive BTK require further investigation.

TLR9, a member of the toll-like receptor family expressed in mammalian immune cells, is a pattern recognition receptor for unmethylated CpG-DNA from bacteria and viruses (41). Ligand-bound TLR9 initiates the production of type I interferons and proinflammatory cytokines to activate host antibacterial or antiviral immune responses (42, 43, 44). Previous studies have reported that BTK was required for TLR9 signaling in monocytic THP1 cells and mouse B cells (45, 46). In addition, proximity labeling experiments revealed the physical association of BCR with TLR9 and MYD88 into a super complex in DLBCL cells (31). These observations suggest that BTK mediates the crosstalk between BCR and TLR9 signaling pathways. Together with our results, the inactivation of BTK kinase activity in DLBCL cells, although not impacting BCR signaling, may weaken the crosstalk between BCR and TLR9 signaling, resulting in increased dependency on *TLR9*. The biochemical and functional interactions between TLR9 and kinase-inactive BTK warrant future studies.

BTKi has transformed the treatment for various B cell malignancies. Thus far, it has been generally believed that BTKi acts by inhibiting the BTK’s catalytic function. Our study raises the intriguing question regarding the exact mechanism of action of BTKi. BTKi such as ibrutinib not only inactivates BTK kinase activity but also shuts down oncogenic BCR signaling. Given our finding that BTK kinase activity is dispensable for oncogenic BCR signaling, we propose that BTKi in clinical use may target BCR signaling by impairing BTK’s noncatalytic function. Collectively, our findings set the stage for studying BTK’s noncatalytic functions in various forms of B cell malignancies.

## Experimental procedures

Recombinant protein purification, cell line engineering, Western blotting, proteomic methods, and chemical synthesis are described in Supplementary Methods. Sources of cell lines, antibodies, plasmids, and compounds are described in Table S1. All human lymphoma cell lines were cultured in RPMI-1640 medium with 10% fetal bovine serum and 2 mM L-glutamine and were confirmed to be *mycoplasma* free on a weekly basis using a PCR-based assay.

### Thermal shift assay

Recombinant BTK kinase domain was diluted to 2 μM with the assay buffer (50 mM Tris pH 7.4, 10 mM MgCl_2_, and 2 mM MnCl_2_). ATP (1 mM) was added to the diluted protein. Twenty microliters of protein–ATP mix was combined with 5 μl of 1:200 diluted SYPRO Orange Protein Gel Stain (Sigma-Aldrich). After incubation on ice for 20 min, fluorescence measurements were performed using a CFX96 Touch Real-Time PCR instrument (Bio-Rad Laboratories, Inc). The temperature was increased from 10 °C to 95 °C with an increment of 0.5 °C and equilibration time of 10 s at each temperature prior to measurement. The melting temperature (Tm) was defined as the temperature corresponding to the maximum value of the first derivative of fluorescence transition.

### *In vitro* BTK kinase assay

Recombinant BTK kinase domain (1 μM) and PLCγ2 peptide (40 μM) or recombinant BTK SH3 domain (40 μM) were diluted with the kinase assay buffer (25 mM Tris pH 7.5, 150 mM NaCl, 5% glycerol, 20 mM MgCl_2_, and 1 mM DTT) and mixed with ATP (50 μM). Reactions (20 μl) were incubated at room temperature for 0, 2.5, 5, 10, 20, 40, and 60 min. ADP level was measured using the ADP-Glo Kinase Assay kit (Promega Corporation). Luminescence was recorded by EnSpire multimode reader (PerkinElmer Inc). Relative ADP level was determined with GraphPad Prism (https://www.graphpad.com/scientific-software/prism/) using baseline correction (by normalizing to 0 min).

### pY proteomics

A total of 5 × 10^7^ cells were treated with 10 nM ibrutinib for 6 h followed by a 5-min stimulation with 10 μg/ml anti-IgM (Jackson ImmunoResearch Laboratories, Inc; 109-006-129). Procedures of deep pY proteomics are described in Supplementary Methods.

### Ca^2+^ flux measurement by flow cytometry

Cells were rinsed three times with Dulbecco's phosphate-buffered saline (DPBS) and incubated in the dark with 1 μM Fluo-4-AM (Beyotime Inc; S1060) diluted in DPBS at 37 °C for 30 min. Dye-loaded cells were washed three times and resuspended in DPBS for an additional 20-min incubation at room temperature. Fluorescence measurements were performed using a BD Accuri C6 Plus flow cytometer (BD Biosciences) using an air-cooled argon ion laser (488 nm excitation). Stimulation with 10 μg/ml anti-IgM (Jackson ImmunoResearch Laboratories, Inc) was performed by injection with a syringe. For data analysis, the relative fluorescence units were normalized as (F − F_0_)/F_0_, in which F_0_ was defined as the mean of fluorescence 5 to 10 s before anti–IgM stimulation. F was defined as the average fluorescence intensity per second. Areas under the calcium flux curves were determined with GraphPad Prism.

### RNA-seq and gene ontology analysis

Total cellular RNA was purified from cells treated with vehicle (dimethyl sulfoxide) or 10 nM ibrutinib for 24 h. Standard RNA-seq was performed by Berry Genomics. Sequencing reads were aligned to the human GRCh38 reference transcriptome using Botwie2 (47) followed by gene-level quantification with RSEM (48). Differential gene expression analyses were performed with DESeq2 (49) with the following cutoff: absolute log_2_-transformed fold change greater than 2 and *p*-value less than 0.01. Gene ontology analysis of differentially expressed genes was performed with clusterProfiler (50).

### Competitive cell growth assay

Cas9-transduced cell lines were infected with the indicated sgRNA lentivirus at a low multiplicity of infection (MOI = 0.2–0.4). Percentages of transduced cells (sgRNA^+^) marked by mNeonGreen-2A-ZsGreen1 were quantified every 2 to 3 days using BD Accuri C6 Plus flow cytometer (BD Biosciences) and normalized to day 4 or 5.

### Parallel genome-wide CRISPR-Cas9 screening

The human CRISPR Brunello library (51) was transduced into TMD8-Cas9 and TMD8-Cas9 *BTK* L528W knock-in cells at a low multiplicity of infection (MOI = 0.2–0.3) and a coverage of ∼400 cells per sgRNA. After puromycin (1 μg/ml) selection, transduced cells were cultured for 3 weeks. Library preparation for sequencing was carried out in PCR performed on genomic DNA isolated from cells. Sequencing reads were analyzed by MAGeCK (52) to determine relative sgRNAs abundance.

### Cell viability assay

Eight thousand cells were plated per well in 96-well microplates (Corning Inc). Cells were treated with serial dilutions of ibrutinib or BGB-15741 with a D300e digital dispenser (Tecan Group Ltd). Cell survival was measured 96 h later using the CellTiter-Glo luminescent cell viability assay kit (Promega Corporation). Luminescence was recorded by EnSpire multimode reader (PerkinElmer Inc). Half maximal inhibitory concentration (IC_50_) was determined with GraphPad Prism using baseline correction (by normalizing to dimethyl sulfoxide control), the asymmetric (five parameter) equation, and least squares fit.

### Statistical analysis

Statistical analyses were performed with GraphPad Prism 8.0. Student’s *t* test was used to evaluate the statistically significant difference between the two sample groups. When comparing more than two independent groups, ANOVA was used to evaluate statistical significance. Multiple comparison tests were performed when ANOVA was significant. All tests were two-tailed, and *p* < 0.05 was considered statistically significant.

## Data availability

Raw data of RNA-seq results have been deposited in the Gene Expression Omnibus (accession number GSE207322). All of the datasets generated during the study are available from the corresponding author upon reasonable request.

## Supporting information

This article contains supporting information (53, 54, 55, 56, 57, 58, 59).

## Conflict of interest

Yutong Zhu, Zhenzhen Cheng, Haimei Xing, and Mike Liu are employees and owning stocks of BeiGene (Beijing) Co, Ltd, Beijing, China. Junjie Hou, Fengjiao Jin, Menglin Li, and Wei Jia are employees and owning stocks of Deepkinase Co, Ltd, Beijing, China. Hongwei Yuan, Yalong Cheng, and Ting Han declare no competing interests.
