# Supplementary material for: BTK kinase activity is dispensable for the survival of diffuse large B-cell lymphoma
Source: J Biol Chem. 2022 Sep 29;298(11):102555. doi: 10.1016/j.jbc.2022.102555 (PMC9636578; doi:10.1016/j.jbc.2022.102555)
Supplement: Supplemental Figures S1–S8 and Tables S1–S5 Legend [file mmc6.pdf]

Supporting information for

BTK kinase activity is dispensable for the survival of diffuse large B-cell lymphoma

Authors:

Hongwei Yuan[1,2], Yutong Zhu[3], Yalong Cheng[1,2], Junjie Hou[4], Fengjiao Jin[4], Menglin Li[4], Wei Jia[4], Zhenzhen Cheng[3], Haimei Xing[3], Mike Liu[3], Ting Han[1,2,5,*]

Affiliations:

[1]College of Life Sciences, Beijing Normal University, Beijing, China.

[2]National Institute of Biological Sciences, Beijing, China.

[3]BeiGene (Beijing) Co., Ltd., Beijing, China.

[4]Deepkinase Co., Ltd, Beijing, China.

[5]Tsinghua Institute of Multidisciplinary Biomedical Research, Tsinghua University, Beijing, China.

***Correspondence:** Ting Han; hanting@nibs.ac.cn

This PDF file includes:

Supplementary Methods

Tables S1-5 (legend)

Figures S1-8

Supplementary Methods

Protein expression and purification

Human BTK kinase domain (residues 389 - 659) was cloned into pFastBac with an N-terminal 6xhis tag. C481S, C481F, C481Y, C481R and L528W mutations were introduced by overlap extension PCR. Production of baculovirus for protein expression in Sf9 cells were performed following routine procedures. Infected Sf9 cells were lysed by sonication in the binding buffer (100 mM Tris-HCl pH 7.6, 300 mM NaCl, 10% glycerol and 2 mM TCEP) supplemented with 2.5 units/ml benzonase (Sigma-Aldrich, St. Louis, MO, USA, F4799) and 2x cOmplete, Mini, EDTA-free protease inhibitor cocktail (Roche, Basel, Switzerland). The lysate was clarified by centrifugation at 15,000 rcf for 1 hour at 4˚C. Recombinant proteins were purified by Ni NTA resin (Smart Lifesciences, Changzhou, China, SA004010) with 30 mM imidazole for washing and 300 mM imidazole for elution. Fractions containing BTK kinase domain were buffer-exchanged using 10 KDa MWCO Amicon ultra centrifugal filters (Merck Millipore, Burlington, NJ, USA) to 100 mM Tris-HCl pH 7.6, 100 mM NaCl, 10% glycerol and 2 mM TCEP, and manually loaded onto a 5 ml HiTrap Q column (GE Healthcare, Chicago, IL, USA). The flow-through containing BTK kinase domain was concentrated, loaded onto ENrich SEC650 size exclusion column (Bio-Rad, Hercules, CA, USA), and then fractionated in 100 mM Tris-HCl, pH 7.6, 100 mM NaCl, and 2 mM TCEP. Fractions containing BTK kinase domain were pooled and concentrated by ultrafiltration, flash frozen with liquid nitrogen, and stored at -80˚C before use.

Human BTK SH3 domain (residues 212-275) were cloned into the pET22b plasmid with a C-terminal 6xhis tag and expressed in E. coli BL21 (DE3). Expression of recombinant BTK

SH3 domain was performed overnight at 18 ℃ in LB medium after induction with 0.5 mM IPTG at an optical density of ~0.8. For protein purification, bacteria were collected and lysed by sonication in the binding buffer (25 mM Tris-HCl pH 7.6, 300 mM NaCl, and 10 mM imidazole) supplemented with 1x cOmplete, Mini, EDTA-free protease inhibitor cocktail (Roche). The lysate was clarified by centrifugation at 15,000 rcf for 1 hour at 4˚C. Recombinant proteins were purified by Ni NTA resin (Smart Lifesciences) with 30 mM imidazole for washing and 300 mM imidazole for elution. Fractions containing BTK SH3 domain were pooled and concentrated, followed by gel filtration on a Superdex 200 10/300 GL column (GE Healthcare) in the gel filtration buffer (25 mM Tris-HCl, pH7.4, 150 mM NaCl). Fractions containing SH3 were pooled and concentrated by a 3 KDa MWCO Ultra centrifugal filter (Merck Millipore), flash frozen with liquid nitrogen, and stored at -80˚C before use.

Lentiviral transduction

Lentivirus was produced in HEK 293T cells by co-transfecting lentiviral vectors with psPAX2 (Addgene, Watertown, MA, USA, 12260) and pMD2.G (Addgene, 12259) in a 5:3:2 ratio. Three days post transfection, cell culture supernatant containing lentivirus was concentrated by sucrose cushion as described (53). In brief, four volumes of cell culture supernatant were overlaid on top of 1 volume of sucrose cushion (50 mM Tris-HCl pH 7.4, 100 mM NaCl, 0.5 mM EDTA and 10% sucrose (m/v)) and centrifuged at 10,000 rcf, 4 ℃ for 4 hours. After centrifugation, the supernatant was carefully removed and ice-cold phosphate buffered saline (PBS) was used to solubilize pelleted virus at 4 ℃ overnight. For viral

transduction, cells were spinfected with concentrated lentivirus at 34 ℃, 1,000 rcf for 2 hours. After spinfection, medium with lentivirus was replaced with fresh medium. Three days post spinfection, cells were selected by antibiotic resistance encoded on the lentiviral vectors.

BTK cDNA expression

UBC promoter was chosen to drive BTK expression after testing a panel of commonly used promoters. Full-length BTK cDNA was cloned into the lentiviral vector Lenti-Ubc-hPGK-Blast. Stable cell lines were generated by lentiviral transduction and blasticidin selection.

Quantitative phospho-tyrosine proteomics by LC-MS/MS

The collected cell pellets were lysed by ultrasonication in the lysis buffer (50 mM Tris-HCl pH7.4, 8 M urea, 1% Triton X-100) freshly supplemented with 0.5% protease cocktail (Merk Millipore, #539134, v/v) and 1% phosphatase inhibitor (Merk Millipore, #524625, v/v). The lysate was clarified by centrifugation at 15,000 rcf for 1 hour at 4˚C. The supernatant was collected and the protein concentration was determined with BCA kit (Thermo Fisher, Waltham, MA, USA) according to the manufacturer's instructions. Six hundred µg of protein per sample was reduced with 8.8 mM dithiothreitol for 1 hour at room temperature and alkylated with 35 mM iodoacetamide for 1 hour at room temperature in the dark. Protein digestion was performed using sequencing grade modified trypsin (Promega, Madison, WI, USA) following a single-pot solid-phase-enhanced sample preparation (SP3) method (54).

Two hundred micrograms of peptides per sample were aliquoted, vacuum-dried, and

resuspended in 1 ml of IAP buffer (50 mM Tris-HCl, 50 mM NaCl, 10 mM NaH_2PO_4, pH 7.6). The phospho-tyrosine peptides were enriched by the SH2 superbinder with IAP buffer and 50 mM ammonium bicarbonate for washing, followed by 0.5% trifluoroacetic acid (TFA) for elution. Afterwards, samples were desalted by C18 SPE column (Phenomenex, Torrance, CA, USA) and dried by vacuum centrifugation.

All nano-LC-MS/MS experiments were conducted on a Orbitrap Exploris 480 (Thermo Fisher) equipped with an Easy n-LC 1200 HPLC system (Thermo Fisher). The peptides were loaded onto a 100 μm id×2 cm fused silica trap column packed in-house with reversed phase silica (Reprosil-Pur C18 AQ, 5 μm, Dr. Maisch GmbH) and then separated on an a 75 μm id×20 cm C18 column packed with reversed phase silica (Reprosil-Pur C18 AQ, 3 μm, Dr. Maisch GmbH). The peptides bounded on the column were eluted with a 73-min linear gradient. The solvent A consisted of 0.1% formic acid in water solution and the solvent B consisted of 80% acetonitrile and 0.1% formic acid. The segmented gradient was 4–9% B, 3 min; 9–20% B, 22 min; 20–30% B, 20 min; 30-40% B, 15 min; 40-95% B, 3 min; 95% B, 10 min at a flow rate of 300 nl/min. With the data-dependent acquisition mode, the MS data were acquired at a high resolution 60,000 (m/z 200) across the mass range of 350–1500 m/z. The target value was 3.00E+06 with a maximum injection time of 22 ms. Data dependent mode was selected as cycle time mode with a duration of 2 seconds. The precursor ions were selected from each MS full scan with an isolation width of 1.6 m/z for fragmentation at normalized collision energy of 28%. Subsequently, MS/MS spectra at resolution 15,000 at m/z 200 were acquired. The target value was 7.50E+04 with a maximum injection time of 22 ms. The dynamic exclusion time was 40 s. For nano electrospray ion source setting, the spray

voltage was 2.0 kV. There was no sheath gas flow and the heated capillary temperature was 320 ℃. Two compensation voltages (CVs) are set as -45 V and -60 V, in order to pass different groups of ions through the high field asymmetric waveform ion mobility spectrometry (FAIMS) interface to the mass spectrometer. The raw MS/MS data were processed using MSFragger version 3.4 (55) against the human Uniprot sequences (20600 entries, one protein sequence per gene). Trypsin (full) was specified as cleavage enzyme allowing up to 2 missing cleavages. The mass tolerance for both precursor ions and fragment ions was set as 20 ppm. Carbamidomethyl on Cys was specified as fixed modification, phosphorylation on Ser/Thr/Tyr, oxidation on Met and acetylation on protein N-term were specified as variable modifications. Proteins were quantified using label-free quantification tool IonQuant with FDR-controlled match-between-runs (56). Both protein and peptides were adjusted to FDR < 1%. Phosphorylation sites were localized with confidence score above 0.75. All the other parameters in FragPipe 17.1 were set to default values.

Western blotting

Standard SDS-PAGE and western blotting procedures were used with the following modifications. For preparation of total lysates, cells were rinsed with DPBS to remove residual medium and then lysed in 20 mM HEPES-NaOH pH 8.0, 10 mM NaCl, 2 mM $MgCl_2$, 1% SDS freshly supplemented with 1 units/ml of benzonase, 1x cOmplete, Mini, EDTA-free protease inhibitor cocktail (Roche), 1x PhosSTOP EASYpack (Roche) and 1 mM pervanadate (Bioss, Beijing, China, D50428). Protein concentrations of the lysates were quantified by the BCA method. For blotting total and phospho-protein, 10–30 μg and 50–100

μg of protein was resolved on SDS-PAGE and transferred to nitrocellulose membranes with a pore size of 0.5 μm. Membranes were blocked in 5% nonfat milk PBST (0.1% v/v Tween-20) for 30 min before blotting with antibodies. The following primary antibodies were used by dilution in 5% nonfat milk PBST: anti-BTK (Cell Signaling Technology, Danvers, MA, USA, 8547S, 1:4,000), anti-PLCγ2 (Cell Signaling Technology, 3872S, 1:1,000), anti-phosphoTyr223-BTK (Cell Signaling Technology, 87141S, 1:1,000), anti-phosphoTyr759-PLCγ2 (Thermo Fisher, PA5-105761, 1:1,000), anti-phosphoTyr1217-PLCγ2 (Cell Signaling Technology, 3871S, 1:1,000), and anti-β-actin-HRP (Huaxingbio, Beijing, China, HX18271, 1:10,000). The following HRP-linked secondary antibodies were used by dilution in PBST: anti-rabbit IgG (Cell Signaling Technology, 7074S, 1:10,000) and anti-mouse IgG (Zsbio, Beijing, China, ZB-2305, 1:10,000). M5 HiPer ECL Western HRP Substrate (Mei5bio, Beijing, China, MF074-01) was used for the detection of HRP enzymatic activity. Western blot images were taken with a VILBER FUSION FX7 imager.

Knock-in of *BTK* L528W in TMD8 cells

In vitro transcribed sgRNA targeting *BTK* (5'-GUGCCUUUAACCUCUGUGCUguuuuagagcuagaaauagcaaguuaaaauaaggcuaguccguuaucaacuugaaaaaguggcaccgagucggugc-3') was mixed with recombinant Cas9 protein (GenScript, Nanjing, China) to form a Cas9-sgRNA ribonucleoprotein complex at 37 ℃ for 10 minutes. For *BTK* L528W knock-in, four million of TMD8 cells were nucleofected in a 4D-Nucleofector (Lonza, Basel, Switzerland) with Cas9-sgRNA complex and a donor

plasmid containing the following repair template:

5’-TTCTACTGGTCAGCAGAAGCTTTGTcCCctgACaagcGTcCTGGGGACGGAGTCTC ACTGGTCTCTGTTTGCACTACAGGCAGCTCGAAACTGTTgGGTAAACGATCAAGG AGTTGTTAAAGTATCTGATTTCGGCCTGTCCAGGTGAGTGTGGCTTTTTCATCTTTC CCTC-3’. Forty-eight hours after nucleofection, cells were seeded in 96-well plates to allow clone formation. Clones were isolated, expanded, and screened for L528W genomic editing via Sanger sequencing.

Generation of cell lines with high Cas9 activity

SFFV promoter was cloned into lentiCas9-Blast (Addgene, 52962) to replace its EFS promoter. The resulting plenti-SFFV-Cas9-Blast plasmid was packaged into lentivirus, which used to transduce human B lymphoma cells. Stable cell lines were obtained by selection with 10 μg/ml blasticidin. For Cas9 clone generation, isolated single clones were tested for their Cas9 activity after transduction with a *POLD3*-targeting sgRNA. Clones with high Cas9 activity were selected based on the kinetics of depletion of sgRNA$^+$ cells.

Quantitative Mass Spectrometry

Parental TMD8 cells were treated with 40 nM BGB-15741 or DMSO for 6 hours in triplicates, followed by quantitative proteomics analysis (PTM BIO, Hangzhou, China). In brief, cells were rinsed with cold DPBS to remove residual medium and then lysed under denaturing conditions (8 M urea freshly supplemented with 1% protease inhibitor cocktail). Samples were sonicated three times on ice using a high intensity ultrasonic processor (Scientz,

Ningbo, China) in lysis buffer (8 M urea, 1% protease inhibitor cocktail). The remaining debris was removed by centrifugation at 12,000 rcf at 4 ℃ for 10 minutes. The supernatant was collected and the protein concentration was determined with BCA kit according to the manufacturer's instructions. For trypsin digestion, protein solution was reduced with 5 mM dithiothreitol for 30 minutes at 56 ℃ and alkylated with 11 mM iodoacetamide for 15 minutes at room temperature in the dark. Protein samples were then diluted by adding 100 mM TEAB to reduce urea concentration to <2 M. Trypsin was added at 1:50 trypsin-to-protein mass ratio for the first digestion overnight and 1:100 trypsin-to-protein mass ratio for a second 4-hour digestion. Afterwards, samples were desalted by C18 SPE column (Phenomenex) and reconstituted in 500 mM TEAB. Peptides were labelled by tandem mass tag (TMT) 6-plex reagent (Thermo Fisher, # 90061) at room temperature for 2 hours. Five microliters of each sample were pooled, desalted and analyzed by mass spec to check labeling efficiency. Afterwards, samples were quenched by 5% hydroxylamine (final concentration), pooled together and desalted with Strata X C18 SPE column (Phenomenex) and dried by vacuum centrifugation.

Labeled peptides were fractionated into fractions by high pH reverse-phase HPLC using Agilent 300 Extend C18 column (5 μm particles, 4.6 mm ID, 250 mm length). Briefly, peptides were separated with a gradient of 8% to 32% acetonitrile in 10 mM ammonium bicarbonate pH 10 over 60 min into 60 fractions. Then, the peptides were combined into 6 fractions and dried by vacuum centrifuging. Fractionated peptides were dissolved in solvent A (0.1% formic acid, 2% acetonitrile/ in water) and directly loaded onto a home-made reversed-phase analytical column (25-cm length, 75 μm i.d.). Peptides were separated with a

gradient from 7% to 11% solvent B (0.1% formic acid in 90% acetonitrile) over 4 minutes, 11% to 32% in 39 minutes and climbing to 80% in 4 minutes then holding at 80% for the last 4 minutes, all at a constant flowrate of 500 nL/min on an EASY-nLC 1200 UPLC system (Thermo Fisher).

The separated peptides were analyzed in Orbitrap Exploris™ 480 (Thermo Fisher) with a nano-electrospray ion source. The electrospray voltage applied was 2.3 kV. The full MS scan resolution was set to 60,000 for a scan range of 400–1200 m/z. Up to 25 most abundant precursors were then selected for further MS/MS analyses with 30 second dynamic exclusion. The HCD fragmentation was performed at a normalized collision energy (NCE) of 35%. The fragments were detected in the Orbitrap at a resolution of 15,000. Fixed first mass was set as 100 m/z. Automatic gain control (AGC) target was set at 5E4.

The resulting MS/MS data were processed using Proteome Discoverer (v2.4.1.15). Tandem mass spectra were searched against the Homo_sapiens_9606_SP_20210721.fasta (20387 entries) concatenated with reverse decoy database. Trypsin/P was specified as cleavage enzyme allowing up to 2 missing cleavages. The mass tolerance for precursor ions was set as 10 ppm in first search and 5 ppm in main search, and the mass tolerance for fragment ions was set as 0.02 Da. Carbamidomethyl on Cystine and TMT6plex (peptide N-Terminus), TMT6plex (K) was specified as fixed modification, and acetylation on protein N-terminal and oxidation on Met were specified as variable modifications. FDR was adjusted to < 1%.

Synthesis of BGB-15741

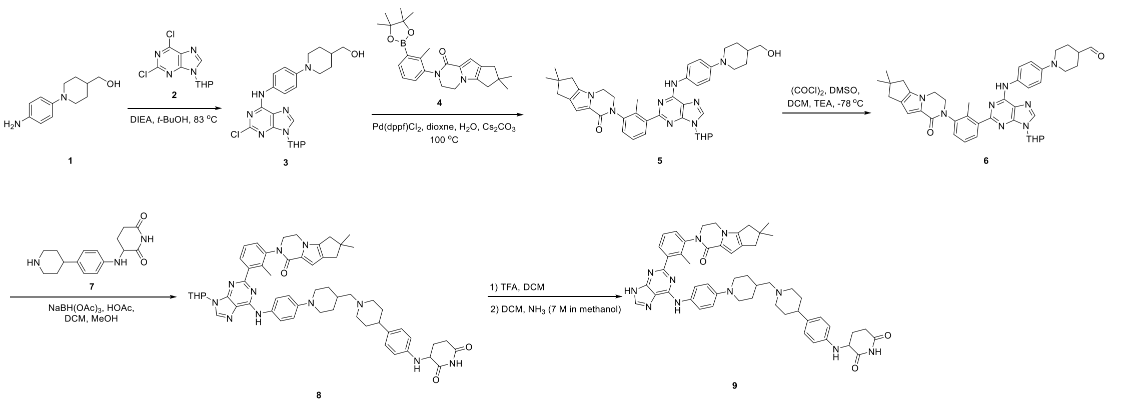

General. All solvents and chemical used were reagent grade. Unless indicated otherwise, the reactions set forth below were performed under a positive pressure of nitrogen or argon or with a drying tube in anhydrous solvents; the reaction flasks were fitted with rubber septa for the introduction of substrates and reagents via syringe; and glassware was oven dried and/or heat dried. ^{1}H NMR spectra were recorded on a Bruker instrument operating at 500 MHz. ^{1}H NMR spectra were obtained using DMSO-d_6 as solvent and residual solvent (DMSO-d_6: 2.50 ppm) as the reference standard. Coupling constants, when given, are reported in Hertz (Hz). LC-MS spectrometer (Agilent 1260) Detector: MWD (190-400 nm), Mass detector: 6120 SQ Mobile phase: A: acetonitrile with 0.1% formic acid, B: water with 0.1% formic acid; Column: Poroshell 120 EC-C18, 4.6×50 mm, 2.7 μm; Gradient method: 5-95% B in 1.5 min; 95% B, 0.5 min; 95%-5% B, 2.0-2.1 min; 5% B, 2.1-3.0 min; Flow rate: 1.8 mL/min.

(1-(4-((2-Chloro-9-(tetrahydro-2H-pyran-2-yl)-9H-purin-6-yl)amino)phenyl) piperidin-4-yl)methanol (3). A mixture of 2,6-dichloro-9-(tetrahydro-2H-pyran-2-yl)-9H-purine (2) (57) (1.4 g, 0.5 mmol), (1-(4-aminophenyl)piperidin-4-yl)methanol (1) (58) (1.02 g, 0.5 mmol) and *N,N*-diisopropylethylamine (3.22 g, 2.5 mmol) in *tert*-butanol (20 mL) was stirred at 83°C overnight. Then the resulting mixture was cooled to room temperature and filtered. The solid

was collected to afford 3 (1.3 g, crude), which was used in the next step directly without further purification. ESI-MS calculated for $C_{22}H_{27}ClN_6O_2$ $[M+H]^+$ = 443.2. Found: 443.3.

2-(3-(6-((4-(4-(Hydroxymethyl)piperidin-1-yl)phenyl)amino)-9-(tetrahydro-2H-pyran-2-yl)-9H-purin-2-yl)-2-methylphenyl)-7,7-dimethyl-3,4,7,8-tetrahydro-2H-cyclopenta[4,5]pyrrolo[1,2-a]pyrazin-1(6H)-one (5). A mixture of compound 3 (780 mg, 1.76 mmol), 7,7-dimethyl-2-(2-methyl-3-(4,4,5,5-tetramethyl-1,3,2-dioxaborolan-2-yl)phenyl)-3,4,7,8-tetrahydro-2H-cyclopenta[4,5]pyrrolo[1,2-a]pyrazin-1(6H)-one (4) (1.48 g, 3.53 mmol), Pd(dppf)Cl_2 (129 mg, 0.176 mmol) and cesium carbonate (1.26 g, 3.87 mmol) in dioxane (30 mL) and water (6 mL) was stirred for 2 hour at 100°C under nitrogen atmosphere. Then the resulting mixture was cooled to room temperature and concentrated under reduced pressure. The residue was purified by silica gel column chromatography (petroleum ether: ethyl acetate = 100%:0% to 0%:100%) to afford 5 (1.1 g, 89%). ESI-MS calculated for $C_{41}H_{48}N_8O_3$ $[M+H]^+$ = 701.4. Found: 701.6.

1-(4-((2-(3-(7,7-Dimethyl-1-oxo-1,3,4,6,7,8-hexahydro-2H-cyclopenta[4,5]pyrrolo[1,2-a]pyrazin-2-yl)-2-methylphenyl)-9-(tetrahydro-2H-pyran-2-yl)-9H-purin-6-yl)amino)phenyl)piperidine-4-carbaldehyde (6). Oxalyl chloride (1 M solution) (2.856 ml, 2.856 mmol) was dissolved in dichloromethane (40 mL) and cooled to -78 ℃. Dimethylsulfoxide (445 mg, 5.712 mmol) was added, and the mixture was stirred for 40 min at -78 ℃. Then compound 5 (1 g, 1.428 mmol) dissolved in dichloromethane (10 ml) was added. The mixture was stirred at -78 ℃ for 1 hour. Then triethylamine (865 mg, 8.568 mmol) was added slowly at -78 ℃. The mixture was allowed to warm to room temperature and washed with water. The aqueous phase was extracted with dichloromethane (3 x 50 ml). The combined organic layers were

washed with brine, dried over sodium sulphate, and concentrated to give 6 (950 mg, crude), which was used in the next step directly without further purification. ESI-MS calculated for $C_{41}H_{46}N_8O_3$ $[M+H]^+$ = 699.4. Found: 699.5.

3-((4-(1-((1-(4-((2-(3-(7,7-Dimethyl-1-oxo-1,3,4,6,7,8-hexahydro-2H-cyclopenta [4,5]pyrrolo[1,2-a]pyrazin-2-yl)-2-methylphenyl)-9-(tetrahydro-2H-pyran-2-yl)-9H-purin-6-y l)amino)phenyl)piperidin-4-yl)methyl)piperidin-4-yl)phenyl)amino) piperidine-2,6-dione (8). A mixture of compound 6 (200 mg, crude), 3-((4-(piperidin-4-yl)phenyl)amino)piperidine-2,6-dione (7) (59) (65 mg, 0.217 mmol) in dichloromethane (10 ml) and methanol (10 ml) was stirred at room temperature for 5 mins. Then acetic acid (0.06 ml) was added. The mixture was stirred at room temperature overnight. Then sodium triacetoxyborohydride (302 mg, 1.425 mmol) was added and the mixture was stirred at room temperature for 2 hours. The resulting reaction mixture was concentrated in vacuum, and the residue was purified by silica gel column chromatography (dichloromethane: methanol = 100%: 0% ~ 90%: 10% gradient elution) to give crude product, which was further purified by prep-TLC (dichloromethane: methanol =10: 1) to afford 8 (95 mg, 34%). ESI-MS calculated for $C_{57}H_{67}N_{11}O_4$ $[M+H]^+$ = 970.5. Found: 970.7.

3-((4-(1-((1-(4-((2-(3-(7,7-dimethyl-1-oxo-1,3,4,6,7,8-hexahydro-2H-cyclopenta [4,5]pyrrolo[1,2-a]pyrazin-2-yl)-2-methylphenyl)-9H-purin-6-yl)amino)phenyl) piperidin-4-yl)methyl)piperidin-4-yl)phenyl)amino)piperidine-2,6-dione (9). To a solution of compound 8 (95 mg, 0.103 mmol) in dichloromethane (10 ml), trifluoroacetic acid (2 ml) was added. The mixture was stirred at room temperature overnight, and then concentrated in vacuo. The residue was diluted with dichloromethane (10 ml) and the pH was adjusted to 8

with NH_3 (7 M in methanol). The mixture was stirred at room temperature for 15 mins, then concentrated in vacuo. The residue was purified by prep-TLC (dichloromethane: methanol = 5: 1) to afford 9 (44 mg, 50%). ^{1}H NMR (500 MHz, DMSO) 13.12 (s, 1H), 10.78 (s, 1H), 9.60 (s, 1H), 8.26 (s, 1H), 7.77 (d, *J* = 10.0 Hz, 2H), 7.63-7.55 (m, 1H), 7.33 (d, *J* = 5.0 Hz, 2H), 6.99-6.89 (m, 4H), 6.64 (d, *J* = 5.0 Hz, 2H), 6.49 (s, 1H), 5.76 (s, 1H), 4.28 (s, 1H), 4.22-4.16 (m, 2H), 4.12-4.05 (m, 1H), 3.82-3.72 (m, 1H), 3.69-3.51 (m, 4H), 3.08-2.92 (m, 3H), 2.78-2.70 (m, 1H), 2.70-2.59 (m, 4H), 2.56 (s, 2H), 2.42 (s, 2H), 2.29 (s, 3H), 2.13-2.07 (m, 1H), 1.97 (s, 2H), 1.92-1.82 (m, 5H), 1.41-1.30 (m, 2H), 1.26-1.16 (m, 8H). ESI-MS calculated for $C_{52}H_{59}N_{11}O_3$ $[M+H]^+$ = 886.5. Found: 886.5.

Table S1. Key resource table.

Table S2. Phospho-tyrosine quantification by label-free mass spectrometry.

Table S3. RNA-seq results (gene-level transcripts per million (TPM) counts).

Table S4. TMT-quantification results (DMSO versus BGB-15741 treated TMD8 cells).

Table S5. MAGeCK gene ranking of genome-wide CRISPR-Cas9 screening (parental versus L528W knock-in TMD8 cells).

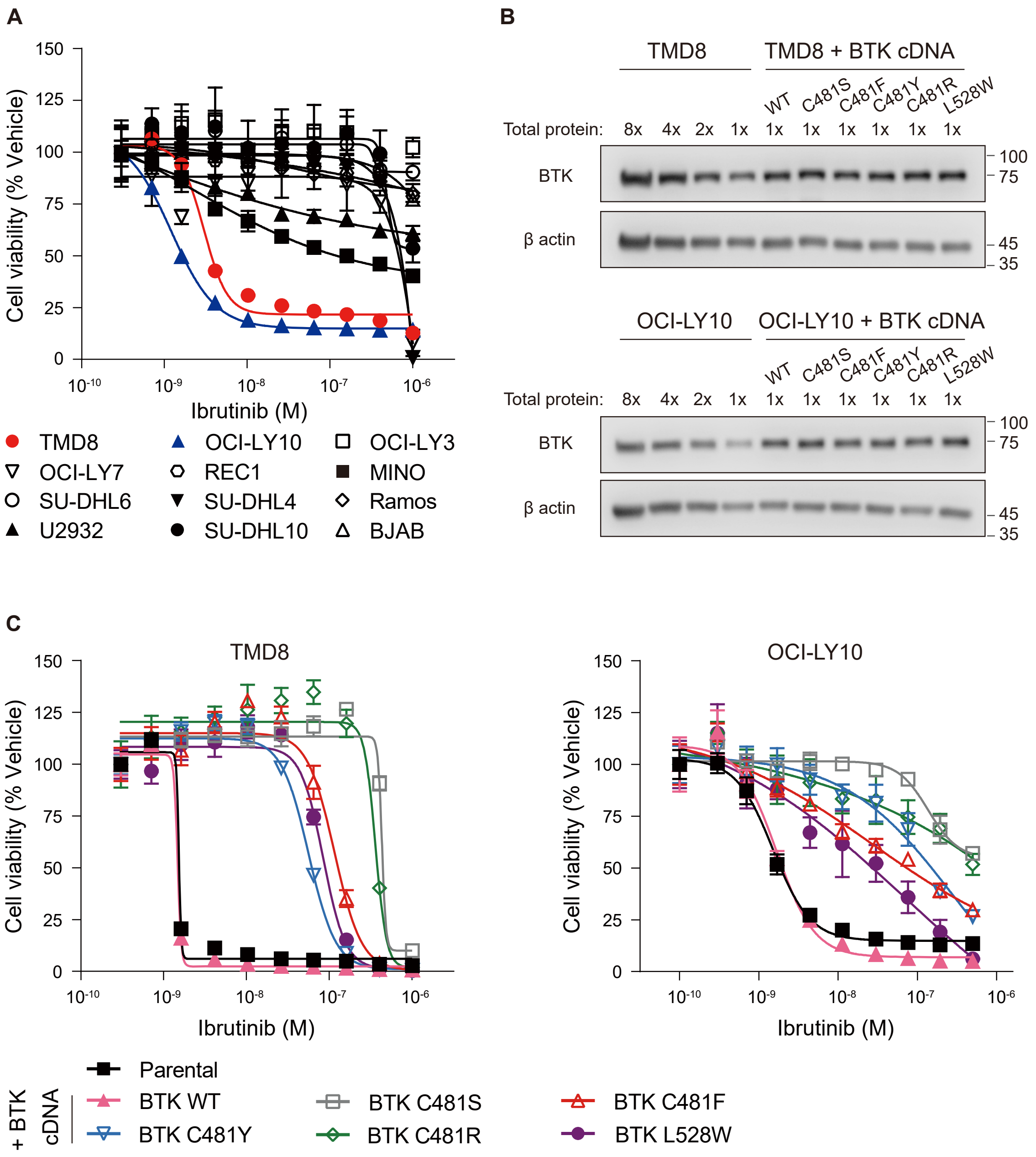

Figure S1. BTK C481S/F/Y/R and L528W confer ibrutinib resistance in DLBCL cells. (A) Ibrutinib cytotoxicity measurements in twelve lymphoma cell lines. (B) Western blotting to measure the level of ectopic BTK expression in TMD8 and OCI-LY10 cells. (C) Ibrutinib cytotoxicity measurements in TMD8 (left) and OCI-LY10 (right) cells expressing BTK wild-type, C481S/F/Y/R and L528W. Data in (A) and (C) are the mean ± SD of three biological replicates.

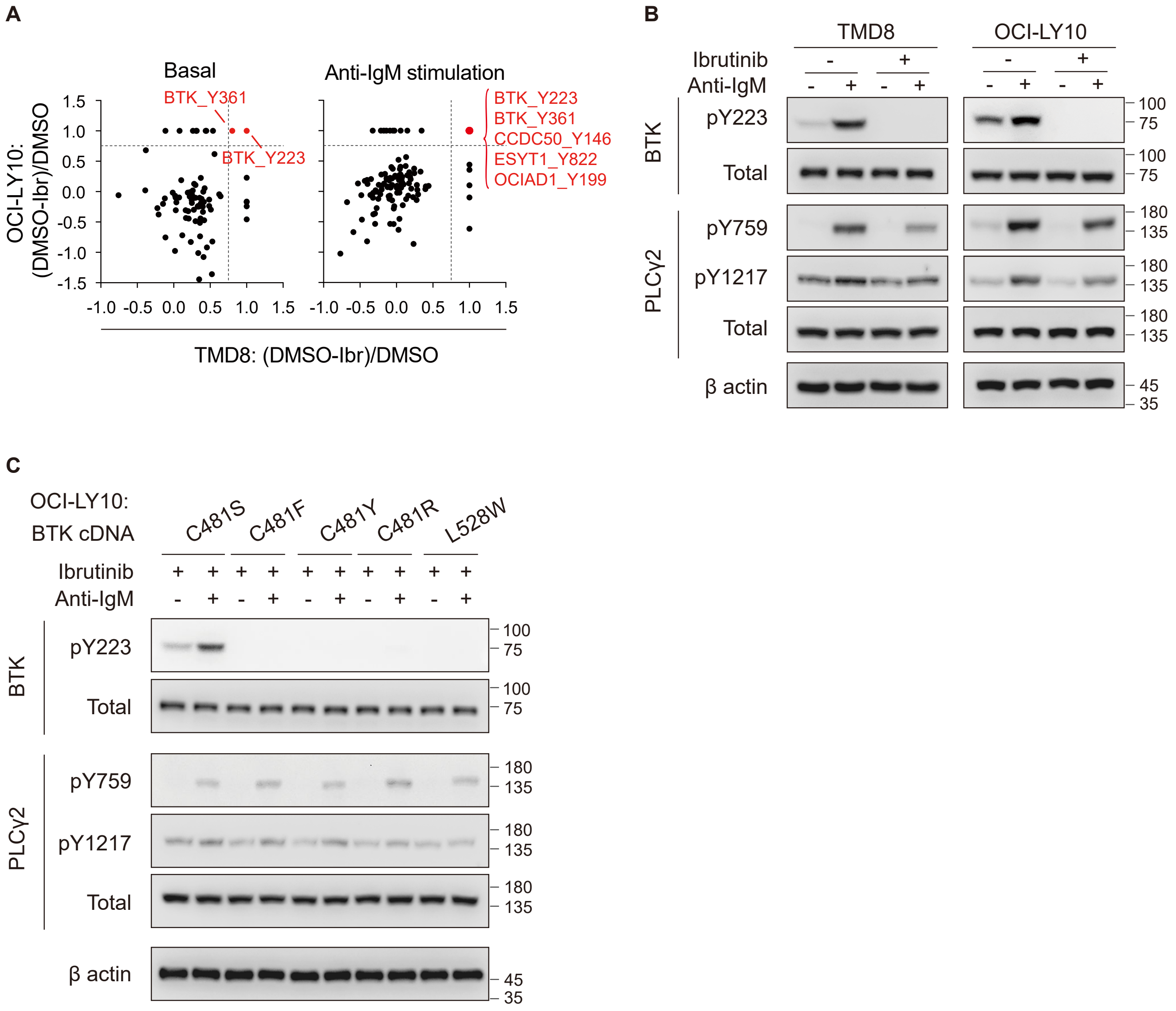

Figure S2. Phospho-tyrosine (pY) proteomics in DLBCL cells. (A) Scatterplots depicting pY proteomic results in parental TMD8 and OCI-LY10 cells under basal condition (left) and anti-IgM stimulation (right). Each dot represents a pY site. The identified sites showing greater than 75% reduction following ibrutinib treatment are colored in red. (B) Western blotting of total and phosphorylated BTK and PLCγ2 in parental TMD8 and OCI-LY10 cells. (C) Western blotting of total and phosphorylated BTK and PLCγ2 in OCI-LY10 cells expressing BTK C481S/F/Y/R and L528W with indicated treatments. Cells were pretreated with 10 nM ibrutinib for 6 hours, followed by 10 μg/ml anti-IgM stimulation.

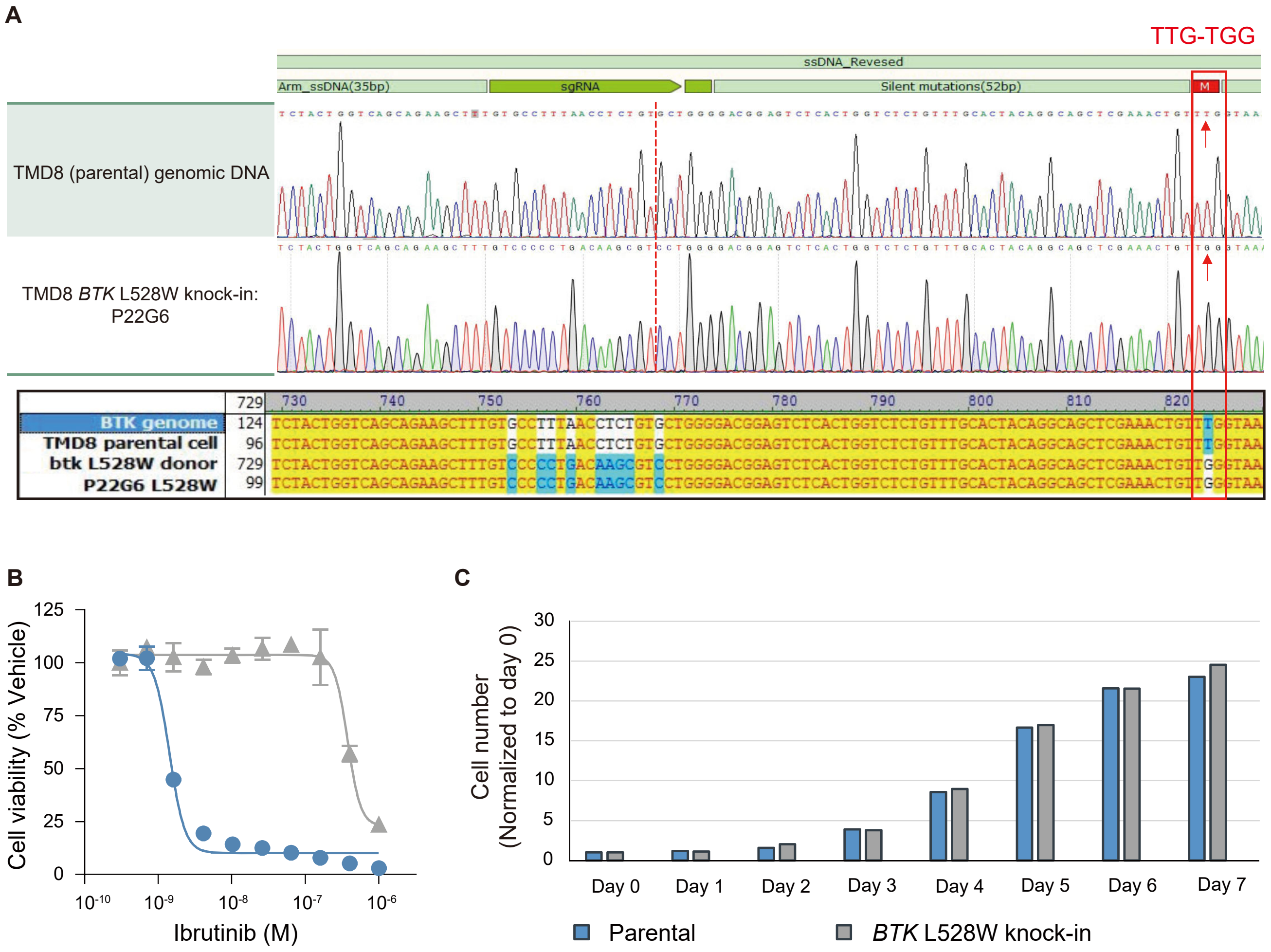

Figure S3. *BTK* L528W knock-in in TMD8 cells. (A) Verification of *BTK* knock-in alleles in TMD8 cells. Target site recognized by sgRNA on the repair template was mutated by synonymous substitutions to prevent sgRNA from cutting. (B) Measurements of ibrutinib IC_{50} in parental (1.42 nM) and *BTK* L528W knock-in (375.4 nM) TMD8 cells. Data are the mean ± SD of three biological replicates. (C) Measurements of cell proliferation rates in parental and *BTK* L528W knock-in TMD8 cells.

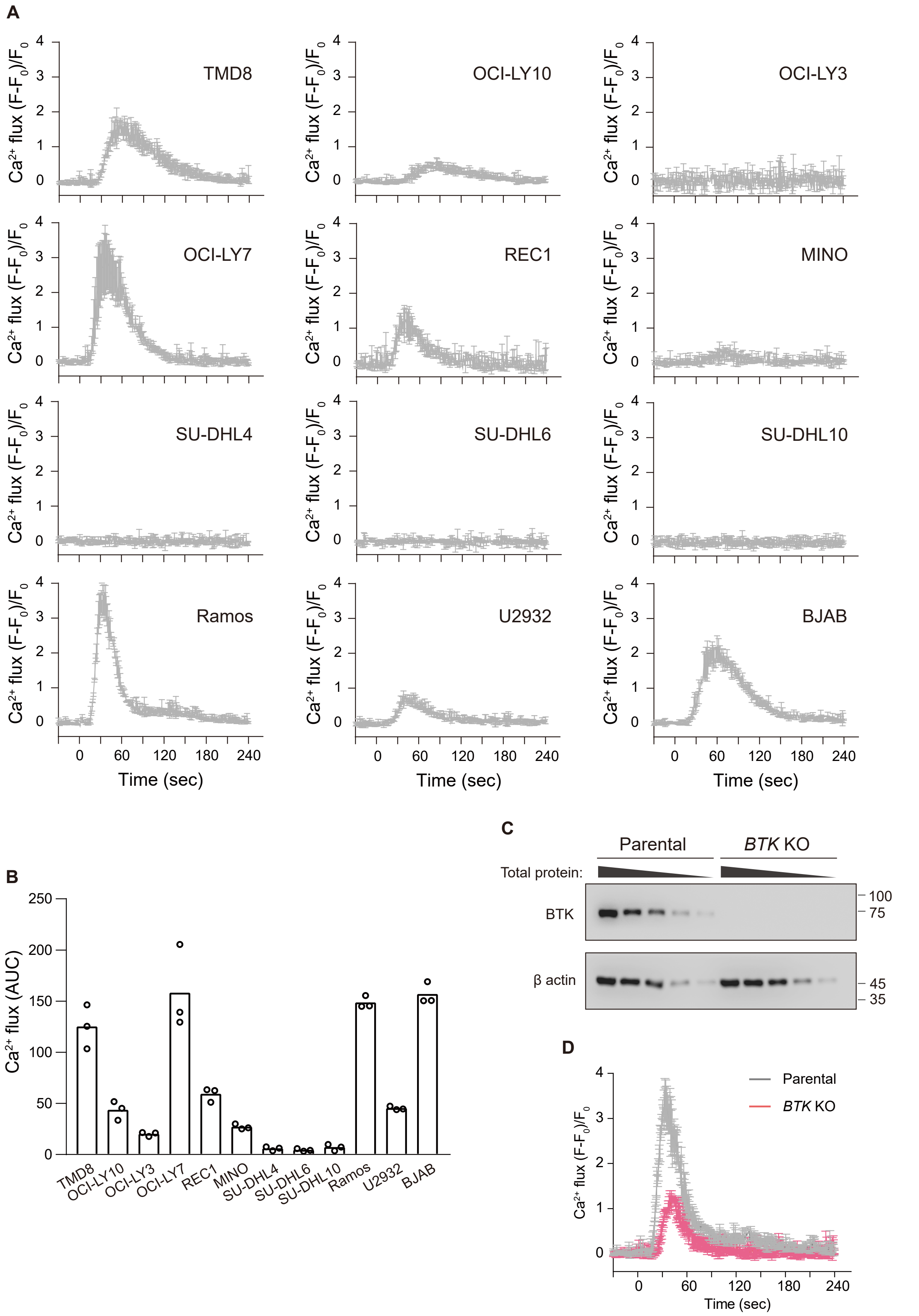

Figure S4. Ca^{2+} flux in malignant B cells. (A) Anti-IgM induced Ca^{2+} flux in twelve lymphoma cell lines. Cells were stimulated with 10 µg/ml anti-IgM. Data are the mean ± SD of three biological replicates. (B) Area under the curve (AUC) quantification of data in (A). (C) Western blot to measure the level of endogenous BTK expression in parental Ramos and a *BTK* knock-out (KO) Ramos clone generated by CRISPR-Cas9. (D) Anti-IgM induced Ca^{2+} flux measurements in parental and *BTK* KO Ramos cells. Data are the mean ± SD of three biological replicates.

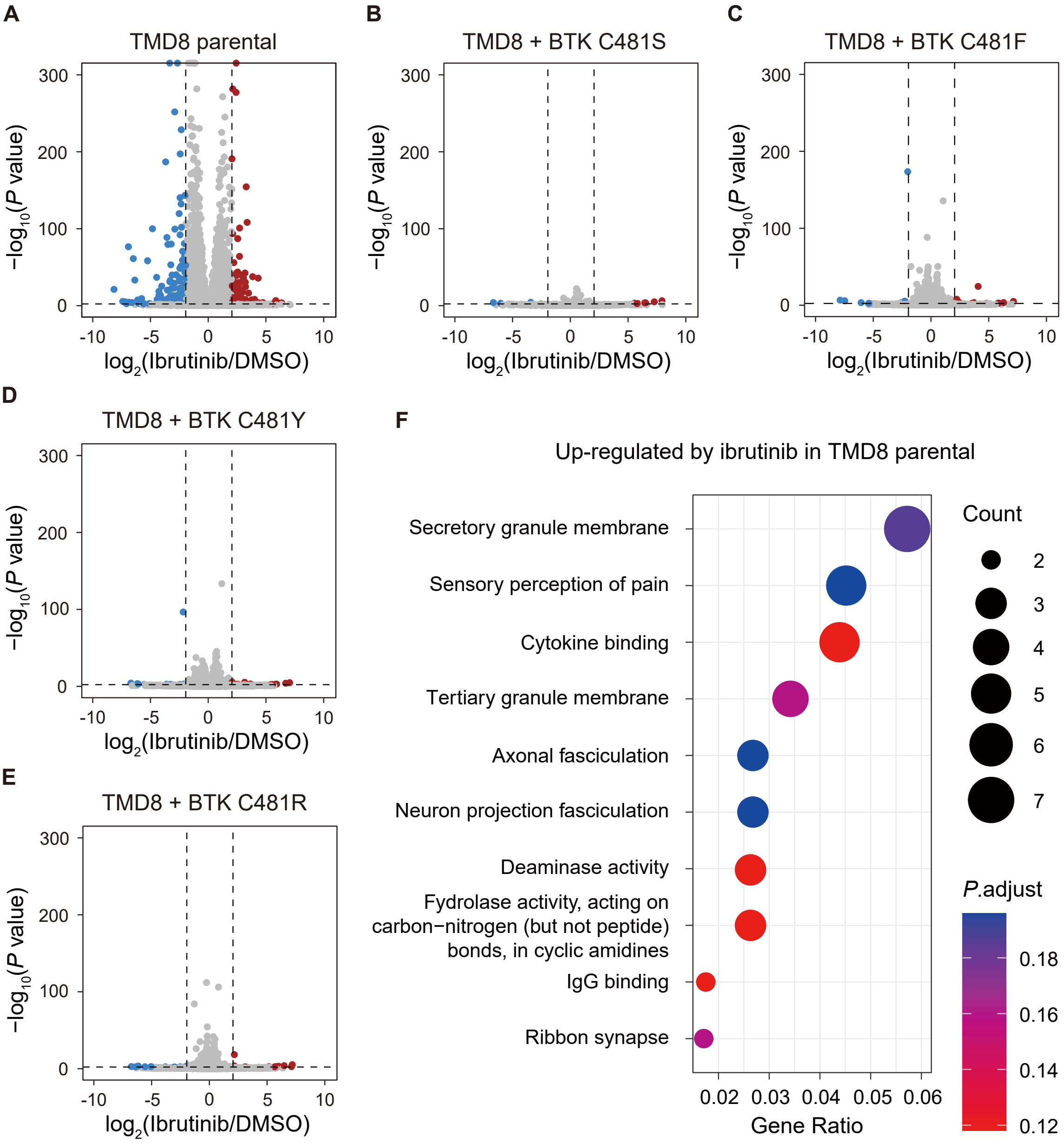

Figure S5. RNA-seq analysis in parental TMD8 cells and TMD8 cells expressing BTK C481S/F/Y/R. (A-E) Volcano plots displaying genes up- or down-regulated by ibrutinib in parental TMD8 cells (A) and TMD8 cells expressing BTK C481S (B), C481F (C), C481Y (D) and C481R (E). (F) Gene ontology enrichment analysis of genes upregulated by ibrutinib in parental TMD8 cells.

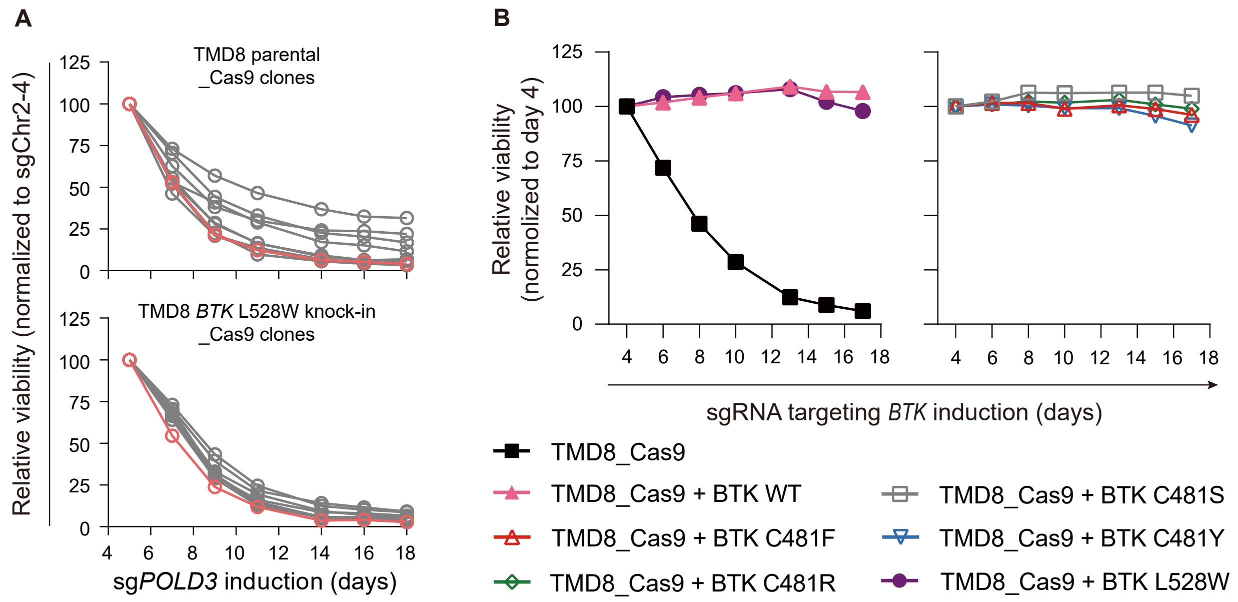

Figure S6. *BTK* genetic dependency analysis in TMD8 cells. (A) Line graphs depicting viability effects after CRISPR inactivation of *POLD3* in different Cas9 clones derived from parental or *BTK* L528W knock-in TMD8. The red traces represent selected clones displaying comparable CRISPR efficiencies. (B) Line graphs depicting viability effects of BTK wild-type, C481S/F/Y/R, and L528W in TMD8 cells after CRISPR inactivation of endogenous *BTK*. Target site on BTK cDNA recognized by sgRNA was mutated by synonymous substitutions to confer sgRNA resistance.

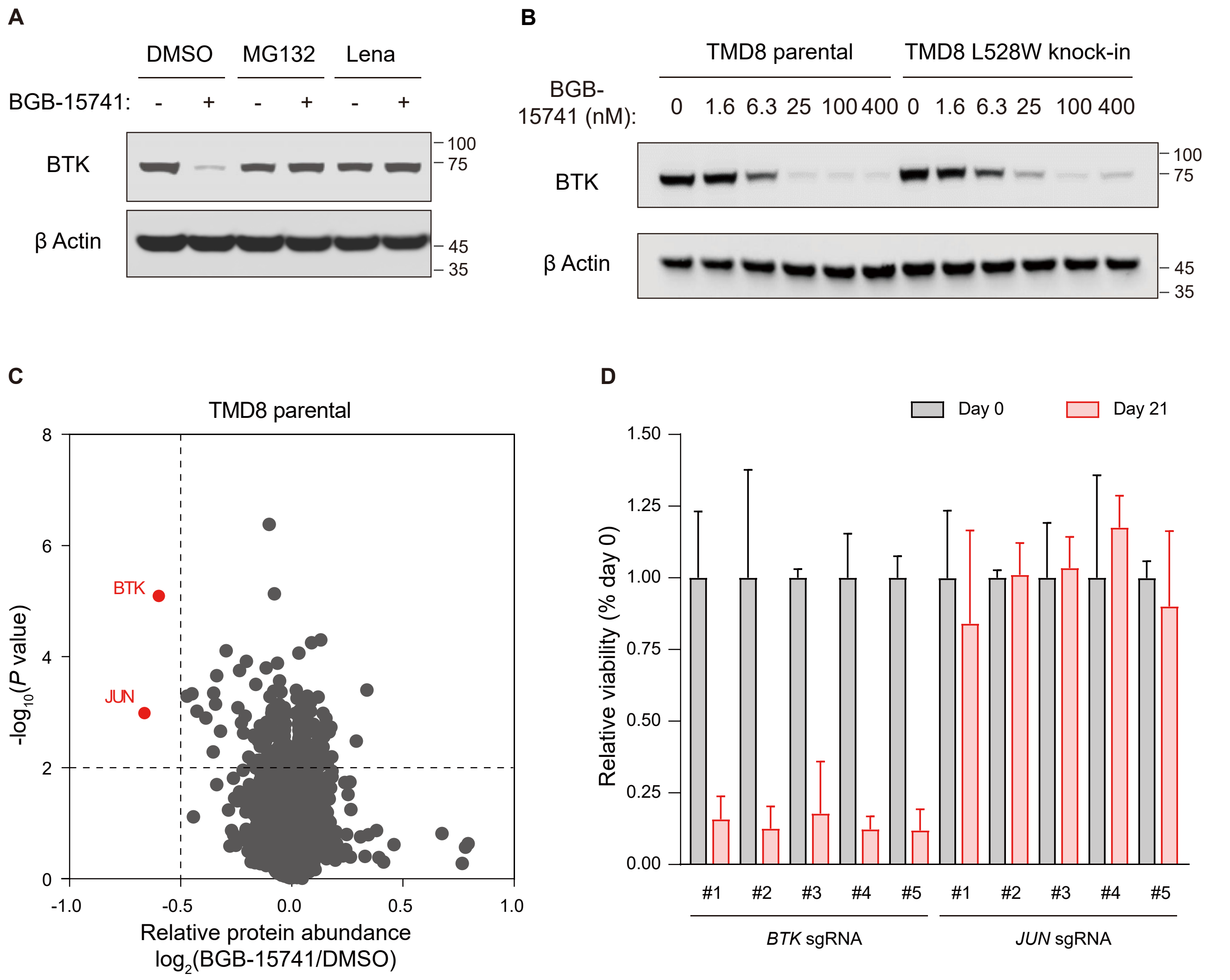

Figure S7. Characterization of BTK PROTAC BGB-15741. (A) Western blotting of MG132 and lenalidomide (Lena) effects on BGB-15741 mediated BTK degradation in parental TMD8 cells. (B) Western blotting of BGB-15741 mediated BTK degradation in parental and *BTK* L528W knock-in TMD8 cells. (C) Scatterplot depicting log2 transformed average fold change in protein abundance and -log10 transformed *P* value in parental TMD8 cells following 40 nM BGB-15741 treatment for 6 hours. Three biological replicates were included in the analyses. (D) Viability effects after CRISPR inactivation of *BTK* and *JUN* in TMD8 cells (data obtained from Phelan *et al.*). Data are the mean ± SD of three biological replicates.

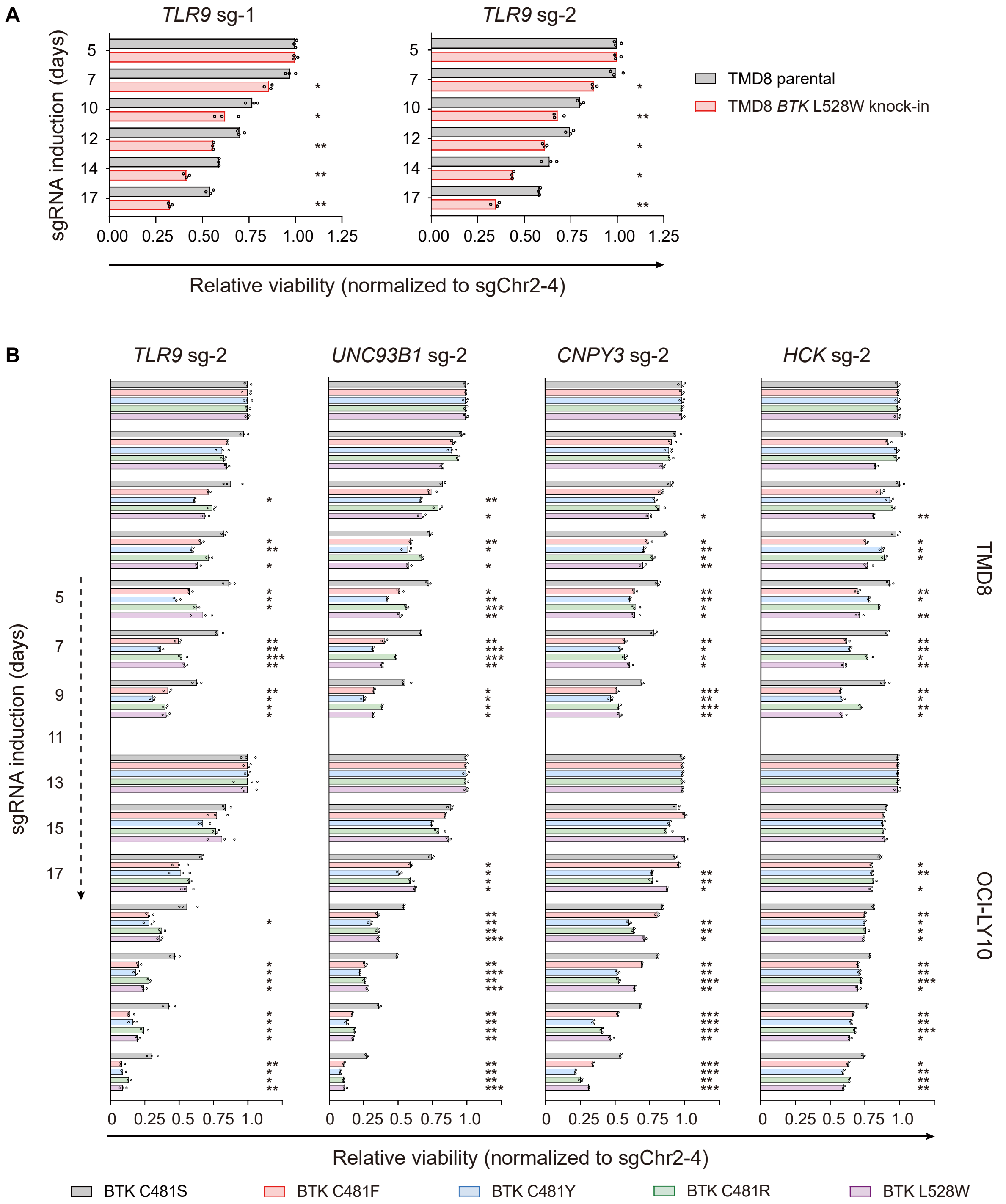

Figure S8. Genetic dependency analysis in DLBCL cells expressing BTK C481S/F/Y/R and L528W.
(A) Viability effects (normalized to the control sgChr2-4) after CRISPR inactivation of *TLR9* in parental and *BTK* L528W knock-in TMD8 cells. Data are the mean of three technical replicates. Comparisons between parental and *BTK* L528W knock-in were analyzed using Student's *t*-test, two-tail, paired. ns: not significant; * $P < 0.05$, ** $P < 0.01$. (B) Viability effects (normalized to the control sgChr2-4) after CRISPR inactivation of indicated genes in indicated cell lines. Endogenous BTK of these cell lines were inactivated by ibrutinib. Data are the mean of three technical replicates from one representative experiment. Two independent experiments were performed. Significance was analyzed using one-way ANOVA with Dunnett's multiple comparison tests (* $P < 0.05$, ** $P < 0.01$, *** $P < 0.001$).
